# Comparative immobilization of labile Pb and Zn fractions in contaminated soil using jackfruit seed-, sugarcane bagasse-, and taro stem-derived biochars: a machine learning-assisted mechanistic elucidation

**DOI:** 10.1039/d6ra01444e

**Published:** 2026-05-06

**Authors:** Truong Xuan Vuong, Ha Ngan Nguyen, The Chinh Pham, Thi Thao Truong, Thi Tam Khieu, Thanh Phuong Phan, Thi Thu Ha Pham, Thi Thu Thuy Nguyen, Xuan Thang Dam

**Affiliations:** a Faculty of Natural Sciences and Technology, Thai Nguyen University of Sciences Tan Thinh Ward Thai Nguyen City 250000 Vietnam xuanvt@tnus.edu.vn; b Faculty of Chemical Technology, Hanoi University of Industry (HaUI) No. 298 Cau Dien Street, Bac Tu Liem District Hanoi Vietnam

## Abstract

Lead (Pb) and zinc (Zn) persist in mining-affected soils due to their association with labile fractions that control mobility and potential bioavailability, necessitating fraction-resolved approaches to evaluate stabilization processes. Biochars derived from sugarcane bagasse, jackfruit seed, and taro stem were produced at 400 °C and applied to contaminated soil at rates of 3%, 5%, and 10% (w/w), followed by a 30 days incubation. Metal fractionation was assessed using the Tessier sequential extraction scheme, coupled with interpretable machine learning. Biochar amendment reduced the exchangeable fraction of both metals and promoted redistribution to less labile pools, with Pb exhibiting a more pronounced shift (up to 61% reduction) than Zn. The extent and direction of redistribution were strongly feedstock-dependent: taro stem biochar preferentially stabilized Pb, whereas jackfruit seed biochar exerted a greater influence on Zn partitioning, demonstrating distinct metal-specific stabilization pathways. Model interpretation using SHAP and partial dependence analysis revealed consistent, metal-specific controls on fraction redistribution, with soil pH, organic carbon, electrical conductivity, and amendment rate emerging as dominant predictors, thereby linking soil chemical conditions to stabilization behavior. Together, these findings indicate that metal stabilization is governed by metal-specific redistribution mechanisms rather than uniform immobilization pathways, providing a quantitative and mechanistically informed framework for optimizing biochar selection in contaminated soils.

## Abstract 2

Lead (Pb) and zinc (Zn) persist in mining-affected soils due to their association with labile fractions that control mobility and potential bioavailability, necessitating fraction-resolved approaches to evaluate stabilization processes.

## Graphical abstract

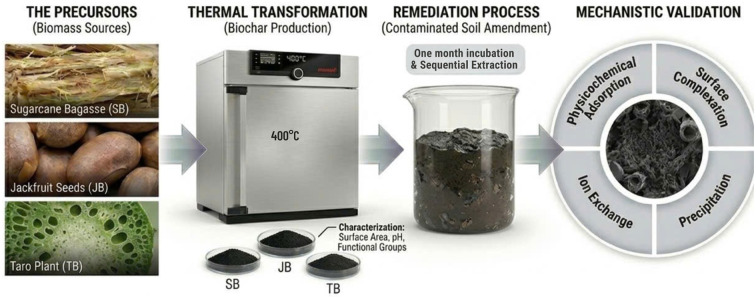

## 1 Introduction

Soil contamination by potentially toxic elements (PTEs), particularly lead (Pb) and zinc (Zn), persists as a long-term environmental constraint because these elements do not degrade and tend to accumulate within terrestrial systems.^1,2^ Their presence in surface soils is closely tied to mining, smelting, industrial discharge, and intensive agricultural inputs, with consequences that extend beyond soil quality to ecosystem functioning and human exposure pathways.^3,4^ This persistence, combined with the potential for remobilization under changing environmental conditions, highlights the need for process-oriented evaluation frameworks rather than static concentration-based assessments. Assessments based solely on total metal concentrations capture only part of this problem. Metal behavior in soils is governed by chemical speciation, which dictates mobility, reactivity, and biological accessibility. Even when total concentrations appear moderate, a substantial proportion in labile forms may sustain ecological risk.^1,2^ Accordingly, fraction-resolved approaches provide a more relevant basis for evaluating stabilization processes under environmentally realistic conditions, particularly in remediation contexts where reductions in total concentration do not necessarily correspond to reduced environmental risk.

The Tessier sequential extraction framework partitions metals into five operationally defined fractions: exchangeable (F1), carbonate-bound (F2), Fe–Mn oxide-associated (F3), organic/sulfide-bound (F4), and residual (F5).^5–7^ This classification enables differentiation between mobile and stable pools, with F1–F2 typically regarded as the most reactive and environmentally relevant. Shifts from these fractions toward more stable forms are commonly interpreted as evidence of immobilization, providing a more informative metric than total concentration alone.^5,8^

Biochar, produced *via* pyrolysis of biomass under oxygen-limited conditions, has been widely explored as a soil amendment for heavy metal stabilization.^9–11^ Its behavior in soil reflects a combination of physicochemical attributes, including alkalinity, carbon structure, electrical conductivity, surface functional groups, and mineral phases. These properties have been associated with multiple processes such as ion exchange, surface complexation, and mineral precipitation in previous work.^12^ However, under realistic soil conditions, the relative contributions of these processes remain difficult to isolate, as multiple factors operate simultaneously and often nonlinearly. In this study, these properties are therefore treated as system-level descriptors rather than explicit predictive variables.

Immobilization mediated by biochar rarely follows a single pathway. Instead, it emerges from interactions between biochar surfaces and soil geochemistry. In alkaline systems, pH elevation can suppress metal solubility, while oxygen-containing functional groups (*e.g.*, –COOH, –OH) may participate in surface binding reactions. Mineral constituents, including carbonate and phosphate phases, introduce additional pathways that influence metal partitioning.^13–15^ These concurrent processes indicate that immobilization is more appropriately interpreted as a system-level outcome rather than the result of a single dominant mechanism.

Findings across the literature do not converge on a uniform outcome. Reduced mobility is frequently reported, but not consistently reproduced under all conditions. In some cases, increases in dissolved organic carbon after biochar addition have been linked to enhanced metal transport.^16^ Partial stabilization of Pb and Zn has also been observed, suggesting that immobilization may remain incomplete under certain soil conditions.^17^ Variability associated with feedstock origin has been repeatedly documented, reflecting differences in ash content, surface chemistry, and mineral composition.^18,19^ Broader syntheses indicate that outcomes range from strongly positive to negligible depending on the environmental setting.^20^ These inconsistencies highlight the lack of a unified framework for resolving how interacting variables govern fraction-level redistribution.

Such divergence points toward a system controlled by interacting variables rather than a single dominant factor. Most studies emphasize bulk removal efficiency or total concentration changes, whereas redistribution among operationally defined fractions receives less consistent attention. This imbalance limits direct comparison across studies and complicates interpretation of stabilization mechanisms. Quantifying the relative influence of controlling variables remains inherently challenging. Soil properties and amendment characteristics often vary concurrently, and available datasets rarely provide sufficient independent observations to isolate individual contributions within a unified analytical framework.^21^ In addition, Pb and Zn are commonly investigated together due to their frequent co-occurrence in contaminated soils; however, differences in their redistribution patterns do not necessarily reflect competitive adsorption processes.^22^ Observations derived from single-soil incubation systems therefore represent co-existing trends under shared conditions rather than direct evidence of surface–scale interactions.

The present study considers three biomass sources, sugarcane bagasse, jackfruit seed, and taro stem, selected for their contrasting lignocellulosic structures, mineral compositions, and regional availability. These inherent differences are known to influence biochar properties following pyrolysis, including porosity, ash content, and surface functional groups, which in turn affect the redistribution behavior of metals within the soil matrix.^2^ By maintaining identical pyrolysis and incubation conditions, this study establishes a controlled comparative framework that enables isolation of feedstock-dependent effects on fraction-level redistribution.

Although the influence of soil parameters such as pH, organic carbon (OC), and electrical conductivity (EC) is widely recognized, their combined and potentially coupled effects on fraction-specific redistribution remain insufficiently resolved in quantitative terms.^23–25^ Data-driven approaches offer a pathway to address this limitation. Machine learning techniques have increasingly been applied in soil–biochar systems to predict adsorption capacity and contaminant removal efficiency;^26–29^ however, these applications are typically focused on bulk performance metrics and do not explicitly resolve how individual soil variables relate to specific geochemical fractions. The key limitation is therefore not predictive capability, but the lack of interpretable frameworks capable of disentangling variable contributions within complex environmental systems.

The novelty of this study lies in establishing a unified and directly comparable analytical framework that explicitly links post-amendment soil properties to fraction-resolved metal redistribution using interpretable machine learning. This approach addresses inconsistencies arising from variability in experimental design and evaluation criteria across existing studies.

The analytical framework is intentionally restricted to variables directly measured following amendment, namely pH, OC, EC, and application rate. These parameters are used as inputs for interpretable machine learning models designed to examine their associations with fraction-resolved redistribution patterns of Pb and Zn. Intrinsic biochar properties, such as surface area and functional group density, are not explicitly parameterized, and no direct attribution is made to these characteristics within the modeling framework.

Conventional regression approaches, whether linear or nonlinear, rely on predefined functional relationships and often have limited capacity to capture nonlinear interactions or coupled effects among soil variables.^26^ In contrast, interpretable machine learning methods provide an alternative by enabling the quantification of variable contributions within complex predictor spaces.^30,31^ Model outputs are therefore interpreted as indicators of statistical association rather than evidence of causality or mechanistic pathways.

Differences observed between Pb and Zn responses are therefore interpreted as comparative patterns under shared environmental conditions, without inferring competitive binding mechanisms or site-specific interactions. Accordingly, the modeling framework supports interpretation of redistribution patterns rather than resolving molecular-scale mechanisms. This work integrates controlled incubation experiments with fraction-resolved geochemical analysis and interpretable modeling. Biochars derived from sugarcane bagasse, jackfruit seed, and taro stem are evaluated under identical pyrolysis and incubation conditions to ensure comparability. The analysis systematically examines how these amendments influence the distribution of Pb and Zn across operationally defined fractions (F1–F5), and how post-amendment soil properties correspond to these changes. Emphasis is placed on identifying dominant influencing factors within the measured variable space, while avoiding overextension into mechanistic interpretations that are not directly supported by the experimental design. This framework may provide a basis for comparing results across studies with differing materials and environmental conditions.

Rather than establishing a predictive or mechanistic model, the objective is to identify dominant variables associated with fraction-level redistribution within the constraints of the measured dataset. Emphasis is placed on interpreting statistically derived relationships while avoiding extrapolation beyond the experimental scope. This positioning helps ensure that conclusions remain consistent with the available data.

**Fig. 1:**
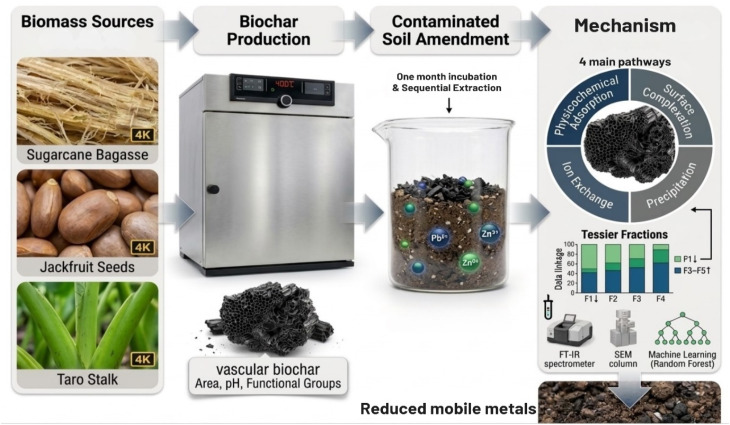
Schematic illustration of the experimental workflow, including biochar production from different biomass feedstocks, biochar characterization, soil amendment and incubation, sequential extraction of Pb and Zn, and the associated immobilization mechanisms.

A schematic overview of the experimental workflow, integrating biochar production, soil incubation, sequential extraction, and mechanistic interpretation, is presented in Fig. 1.

## 2 Materials and methods

### 2.1. Soil sampling and initial characterization

Soil samples were taken from paddy fields in the Pb/Zn mining area of Lang Hich village, which is a region highly susceptible to contamination by potentially toxic elements (PTEs) due to long-term mining activities (21°43′46.27″ N, 105°51′2.75″ E) in the vicinity of a known Pb/Zn mine. Sampling was conducted within a single representative field at this location rather than across multiple independent sites, and five subsampling points were combined to form one homogenized composite sample used as the incubation source material. Specifically, five subsampling points were selected within a 10 × 10 m plot following a diagonal sampling pattern to capture spatial variability. Approximately 0.4 kg of soil was collected from each point and combined to form one composite sample (∼2 kg total). Soil samples were taken at each site from the surface layer, 0–30 cm deep, which corresponds to the actively cultivated soil layer. A total of 2 kg of soil was collected at each sampling point, with a horizontal sampling area of 30 × 30 cm to representatively cover the study area. This composite sample was homogenized and served as the sole source material for all incubation experiments, ensuring consistency across treatments.

After collection, the soil samples were first air-dried at room temperature and then oven-dried at 45 °C for 48 hours.^13^ This was necessary to reduce the effect of moisture variability during the analysis. Visible debris (gravel, roots) was manually removed. The dried soils were homogenized, gently crushed, and sieved (<2 mm).

A subsample was used for baseline physicochemical characterization, including pH, electrical conductivity (EC), organic carbon (OC), particle size distribution, and total Pb and Zn concentrations.^14^ These parameters were later used to interpret biochar-induced changes in soil properties and metal fractionation.

### 2.2. Preparation and characterization of biochars

Biochars from three different locally available biomass wastes, sugarcane bagasse, jackfruit seeds, and taro stalks, were prepared and used to utilize locally available and cheap biomass wastes that have great potential as soil remediation agents. In this study, three locally available agricultural residues, sugarcane bagasse, jackfruit seeds, and taro stalks, have been used as biochar materials, mainly due to their availability and great potential as soil remediation agents from contaminants. By using different biomass wastes that have different compositions of lignocellulosic materials, the effect of biomass composition on biochar properties and PTE immobilization ability can be evaluated. Before undergoing pyrolysis, the raw biomasses were cleaned and washed twice using deionized water and then air-dried, chopped into small pieces, and ground into powder using a grinding machine. Pyrolysis was carried out in a tubular furnace under continuous high-purity nitrogen (N_2_, 99.999%) to maintain oxygen-limited conditions. The system was purged at 3 L min^−1^ for 20 min prior to heating, followed by a constant flow of 1.5 L min^−1^ during carbonization. The temperature was increased to 400 °C at 10 °C min^−1^, held for 60 min, and then allowed to cool naturally under N_2_. The selected temperature preserves oxygen-containing functional groups while ensuring sufficient carbonization for metal binding.

**Fig. 2:**
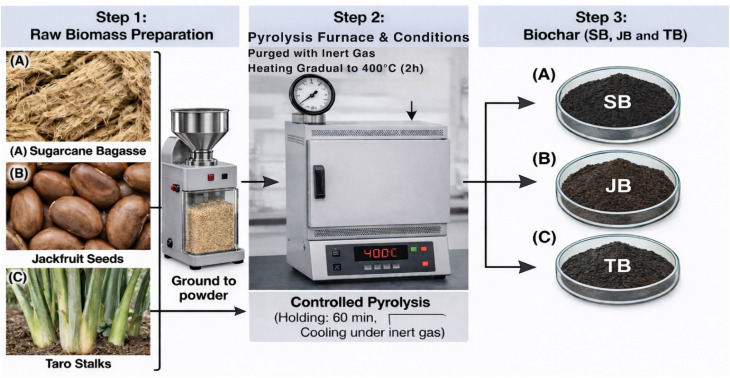
Schematic illustration of biochar production from three different biomass precursors: (A) sugarcane bagasse (SB), (B) jackfruit seeds (JB), and (C) taro stalks (TB). The process involves (step 1) raw biomass preparation followed by grinding into powder, (step 2) controlled pyrolysis under an inert atmosphere with gradual heating to 400 °C and holding for 60 min, and (step 3) collection of the resulting biochars (SB, JB, and TB).

A schematic overview of the preparation process is presented in Fig. 2.

To achieve uniformity of size across all biochars, the original 3 types of biochars (sugarcane bagasse, jackfruit seed, and taro stalks) were ground down to a particle size of <0.5 mm. These biochars were referred to as SB, JB and TB, respectively and stored for later use in sealed polyethylene bags.

The physicochemical properties (pH, electrical conductivity at a water to solid ratio of 1 : 10, BET surface area, pore volume, surface morphology, elemental composition, and surface functional groups) of the produced biochars were characterized to help understand how the properties of biochar interact and immobilize lead (Pb) and zinc (Zn) contamination in the soils.

### 2.3. Physicochemical characterization of biochars

Biochar pH, EC, and organic carbon were determined and used as input variables in the machine-learning (ML) models, whereas BET surface area and O/C ratio were used solely for physicochemical characterization and mechanistic interpretation, and were not included as ML predictors.

• Specific surface area of materials is evaluated using nitrogen adsorption–desorption analysis (BET) measurements. This parameter was used to support interpretation of metal interactions where relevant.

Elemental composition was analyzed to characterize biochar materials.

• Elemental data are reported for general characterization purposes only and are not used as quantitative variables in mechanistic interpretation. Ash-related composition was not included in this study and is therefore not considered in the analysis.

FT-IR spectroscopy was used to identify surface functional groups relevant to metal interaction.

• Functional-group information is interpreted qualitatively to support discussion of interaction mechanisms and is not used as a primary quantitative input.

#### 2.3.1. Basic chemical properties

For the purpose of determining the influence of biochar material characteristics on the pathways by which biochar interacts with soil in relation to lead (Pb) and zinc (Zn), three elemental characteristics of biochar (pH, electrical conductivity (EC), and organic carbon (OC)) were identified as key indicators of potential influence on interaction behavior with Pb and Zn. These parameters were selected because they directly influence soil chemistry and were used in statistical and machine-learning interpretation of metal redistribution. Biochar was mixed with de-ionised water at a 1 : 10 (w/v) ratio and measured for pH and EC in this suspension to achieve stable and reproducible results. The suspensions were gently stirred and allowed to equilibrate before measurement. pH was measured with a Hanna HI 9124 pH meter (Hanna Instruments, Romania), while EC was measured with the built-in conductivity probe.

The organic carbon content (OC) was analysed according to standard methods by using a C/H/N analyzer as outlined in previous studies.^13,32^ Understanding these parameters is of critical importance in determining how specific characteristics of biochar interact with Pb and Zn and influence their immobilisation behaviour in soil.

Elemental composition was analyzed to characterize biochar materials. Only selected elemental data are reported and used to support general material description. Ash-related composition was not included as a quantitative variable in this study.

Surface functional groups were identified using FT-IR spectroscopy, and pore characteristics were examined using BET analysis.

Functional-group information and surface area are interpreted qualitatively to support discussion of interaction mechanisms. They were not used as predictors in the ML models.

#### 2.3.2. Specific surface area and pore structure (BET)

Surface area is important for metal sorption characteristics. The BET surface area analyzer (TriStar II 3020, Micromeritics Instrument Corporation, USA) was used to evaluate the pore structure and specific surface area of biochars based on their values for nitrogen adsorption/desorption isotherms. Prior to conducting the analysis, biochar samples were degassed for 2 hours at 105 °C to obtain accurate results, as failure to degas biochar results in underestimation of surface area.

The linear section of the adsorption isotherm was used with the BET equation to compute the specific surface area.

#### 2.3.3. Fourier-transform infrared spectroscopy (FT-IR)

FT-IR spectroscopy was used to analyse the surface functional groups of the biochars and to provide an idea of how the biochars may interact with Pb and Zn.

The FT-IR spectra were recorded at JASCO FT/IR-4600 (JASCO International Co. Ltd., Tokyo, Japan).

#### 2.3.4. Surface morphology and elemental composition (SEM-EDS)

JSM-6700F FE-SEM and EDS were used to determine the morphology and elemental composition of biochars (JEOL, Tokyo, Japan). This was done to determine surface characteristics that relate to metal retention *via* biochars. SEM was used to provide images of pore development and surface roughness at different magnifications of biochar samples. In addition, EDS was used to document the presence of metals on the surface of biochar as additional support for how metals interact with biochars.

### 2.4. Soil incubation experiment

In this study, a soil incubation experiment was designed to examine how biochar type and application rate influence the immobilization and transformation of labile Pb and Zn fractions in contaminated soil. A total of ten treatments were established, consisting of an unamended control and nine biochar-amended treatments.

The control treatment consisted of untreated soil, while amended treatments involved SB, JB, and TB biochars applied at rates of 3%, 5%, and 10% (w/w). These application rates were intentionally selected to assess dose–dependent effects while maintaining practical relevance for soil remediation.

Each treatment consisted of 100 g dry soil mixed with 3 g, 5 g, or 10 g biochar, corresponding to 3%, 5%, and 10% (w/w), respectively. This explicit mass-based dosing ensures reproducibility and scalability. Soil moisture was adjusted to 70% water-holding capacity and maintained throughout incubation. Samples were incubated at 25–30 °C for 30 days. All treatments were conducted in triplicate (*n* = 3).

Earlier stabilization experiments suggest that most of the redistribution of heavy metals in biochar-treated systems occurs within the first three to four weeks under laboratory conditions. Beyond this window, the partitioning pattern changes little, indicating that a near-equilibrium state has already been established.^13,32^ During this period, soil moisture was regularly monitored and adjusted when necessary as reported in previous studies.^13,32^

After incubation, soils were collected for pH, EC, and Pb/Zn speciation analyses using the Tessier sequential extraction procedure.^13,32^ All treatments were conducted in triplicate (*n* = 3), and treatment codes and experimental design are summarized in Table 1. The soil used in this study was collected from a representative contaminated site and homogenized prior to experimentation to reduce spatial variability. All treatments were conducted in triplicate (*n* = 3) to ensure reproducibility. While this approach allows controlled comparison between treatments, the use of a single soil source may limit the generalizability of the results to other soil types with different physicochemical properties.

**Table 1:** The incubation experiment design^a^

Sample	Sample code	Biochar (g)	Soil (g)	Biochar ratio (%)
Contaminated soil or blank soil (BS)	BS	0	100	0
BS + 3% SB	SB3	3	100	3
BS +5% SB	SB5	5	100	5
BS + 10% SB	SB10	10	100	10
BS+ 3% JB	JB3	3	100	3
BS + 5% JB	JB5	5	100	5
BS + 10% JB	JB10	10	100	10
BS + 3% TB	TB3	3	100	3
BS + 5% TB	TB5	5	100	5
BS + 10% TB	TB10	10	100	10

a BS represents Pb/Zn contaminated soil without biochar amendment (control soil or blank soil). SB3-SB10, JB3-JB10, and TB3-TB10 indicate contaminated soils amended with 3%, 5%, and 10% (w/w) of the sugarcane bagasse, jackfruit seed, and taro stem biochars, respectively.

### 2.5. Tessier sequential extraction procedure and quality assurance/quality control (QA/QC)

Chemical fractionation of Pb and Zn in soil samples was carried out using a modified Tessier sequential extraction scheme, which separates metals into five operationally defined fractions with progressively lower mobility and bioavailability. Sequential extraction was performed independently on each replicate sample prior to averaging, ensuring consistency between experimental and analytical replication. All extraction steps were conducted in triplicate to ensure reproducibility. Sequential extraction followed a modified Tessier protocol, partitioning metals into five operational fractions. These fractions are operationally defined rather than discrete geochemical phases, as partial redistribution or readsorption of metals may occur during extraction due to changes in chemical conditions, such as pH shifts and reagent interactions. We therefore interpret the results as indicative of relative changes in metal mobility rather than absolute speciation. To minimize potential artifacts, all procedures were conducted under strictly controlled conditions, including triplicate extractions and mass balance verification.

For each sample, 1.000 g (dry weight) of air-dried, homogenized soil was transferred into acid-washed polypropylene centrifuge tubes and subjected to the following sequential steps:

• F1 (exchangeable fraction): extracted with 8 mL of 1.0 M MgCl_2_ (pH ≈ 7.0) under continuous shaking for 1 h at room temperature.

• F2 (carbonate-bound fraction): the residue from F1 was treated with 8 mL of 1.0 M sodium acetate (NaOAc), adjusted to pH 5.0 with acetic acid, and shaken for 5 h at room temperature.

• F3 (Fe–Mn oxide-bound fraction): the residue from F2 was extracted with 20 mL of 0.04 M hydroxylamine hydrochloride (NH_2_OH·HCl) in 25% (v/v) acetic acid at 96 ± 2 °C for 6 h, with intermittent agitation.

• F4 (organic matter- and sulfide-bound fraction): the residue from F3 was oxidized with 3 mL of 30% H_2_O_2_ (adjusted to pH 2 with HNO_3_) at 85 ± 2 °C for 2 h, followed by a second identical H_2_O_2_ treatment. After cooling, 5 mL of 1.0 M NH_4_OAc in 20% (v/v) HNO_3_ was added and the mixture was shaken for 30 min.

• F5 (residual fraction): the remaining solid was digested with *aqua regia* (HCl/HNO_3_, 3 : 1, v/v) to quantify metals incorporated within mineral lattices.

After each extraction step, suspensions were centrifuged at 4000 rpm for 30 min. Supernatants were carefully decanted, filtered through 0.45 µm membranes, and preserved at pH < 2 with ultrapure HNO_3_. The solid residues were rinsed once with deionized water, recentrifuged, and the rinse solutions discarded to minimize cross-contamination between steps.

Lead and zinc concentrations in all extracts were quantified by ICP-MS. Procedural blanks were processed alongside samples for each extraction stage and remained negligible relative to sample concentrations.

Quality assurance and quality control were maintained through the use of acid-cleaned labware, multi-element calibration standards, and replicate extractions (*n* = 3). Internal consistency of the sequential extraction was assessed by comparing the cumulative metal content recovered from all fractions (∑F1 − F5) with total metal concentrations obtained from independent *aqua regia* digestion. Recoveries for both Pb and Zn typically ranged between 90% and 110%, indicating acceptable mass balance across the procedure (see Table S1 in SI).

### 2.6. Statistical analysis and machine-learning modeling

#### 2.6.1 Statistics

Principal component analysis (PCA) was conducted as an intermediate step to explore relationships between experimental observations and to support interpretation of machine-learning (ML) results. Input variables included soil-related parameters (pH, organic carbon (OC), electrical conductivity (EC)) and the exchangeable fractions of Pb (F1_Pb) and Zn (F1_Zn). Data were standardized using *z*-score normalization prior to PCA, and components with eigenvalues >1 were retained.

Two-way ANOVA followed by Tukey's HSD test (*p* < 0.05) was used to evaluate the effects of biochar type, application rate, and their interaction.

#### 2.6.2 Machine-learning modeling

The ML modeling dataset included soil pH, organic carbon (OC), electrical conductivity (EC), amendment application rate (3%, 5%, and 10% w/w), and the corresponding Pb and Zn fractions (F1–F5) measured after 30 days of incubation. The objective of the ML analysis was to relate post-amendment soil conditions to the redistribution of Pb and Zn fractions.

Biochar intrinsic properties (*e.g.*, BET surface area and O/C ratio) were not included as predictors in the machine-learning (ML) models and were considered exclusively for physicochemical characterization and mechanistic interpretation. Triplicate measurements (*n* = 3) were averaged prior to ML modeling, such that each row in the dataset represents one independent treatment condition rather than individual replicates. The experimental design comprised 10 treatment conditions, including the control. Pb and Zn fractions were treated as separate response variables and modeled independently, and therefore do not increase the number of independent observations. Accordingly, the final dataset used for ML modeling consisted of *N* = 10 independent observations, each corresponding to a distinct treatment condition.

To enrich model learning while maintaining the same independent structure, additional response variables (Pb and Zn fractions F1–F5) were modeled separately rather than increasing the number of independent observations. Therefore, the dataset size is defined strictly at the treatment level, and no artificial expansion of sample size was performed.

Given the limited dataset size (*N* = 10), ML was explicitly used as an exploratory and hypothesis-generating tool rather than for predictive generalization. We acknowledge that small sample sizes may increase the risk of model instability and overfitting, particularly for flexible nonlinear algorithms.

To mitigate these limitations, several safeguards were implemented. Two complementary ensemble learning approaches, random forest (RF) and extreme gradient boosting (XGBoost), were applied to capture patterns using both bagging- and boosting-based strategies. This dual-model approach enables cross-validation of findings across fundamentally different variance-control mechanisms.

Model performance was evaluated using repeated *k*-fold cross-validation (*k* = 5) with 10 independent repetitions, applied at the level of treatment conditions (rows).

In addition, leave-one-out cross-validation (LOOCV) was used as a complementary validation approach to maximize data utilization under small-sample conditions. All hyperparameters were tuned exclusively within the training folds (nested cross-validation), ensuring that no information leakage occurred.

Model robustness was assessed by comparing training and validation errors and by examining the variability of performance metrics across repeated resampling runs. Metrics were averaged, and their standard deviations were retained to quantify dispersion. From these distributions, 95% confidence intervals for *R*^2^ and RMSE were calculated.

Model performance was evaluated using *R*^2^, root mean square error (RMSE), and mean absolute error (MAE). Residual distributions were examined to identify potential systematic bias, and no consistent deviation across prediction ranges was observed.

Because RF and XGBoost are tree-based algorithms that rely on recursive partitioning rather than distance-based calculations, feature scaling was not applied.

Interpretation of ML outputs relied on SHapley Additive exPlanations (SHAP), which quantify the contribution of each predictor based on cooperative game theory. Both global feature importance and local explanations were analyzed. Partial dependence plots (PDPs) were used to visualize marginal effects of key predictors on Pb and Zn fraction responses.

To ensure robustness, only relationships consistently identified across both algorithms and supported by multiple interpretability tools (SHAP, PDP, and PCA) were considered meaningful. Importantly, interpretation was restricted to variables explicitly included in the ML models (pH, OC, EC, and application rate), while mechanistic insights involving intrinsic biochar properties were derived independently from physicochemical characterization.

All statistical analyses were performed using *R* (version 4.5.2; *R* Foundation for Statistical Computing, Vienna, Austria). Random Forest modeling was conducted using the *randomForest* package, and XGBoost models were implemented using the *xgboost* package. Model interpretability analyses were carried out using the *iml* package. PCA was performed using the *FactoMineR* package and visualized using *factoextra*. Data preprocessing was conducted using *dplyr* and *tidyr*, and graphical outputs were generated using *ggplot2*. RStudio (Posit, Boston, MA, USA) was used as the integrated development environment.

Statistical significance was evaluated at *p* < 0.05, with *P*-values reported for correlation analyses and group comparisons, and significance levels indicated in figures using appropriate annotations. Potential sources of variability include soil heterogeneity, variability in biochar–soil interactions, and uncertainties associated with sequential extraction procedures. These factors were considered in the interpretation of multivariate relationships, and conclusions were not based on single-factor correlations.

Potential sources of variability include heterogeneity in soil properties, variability in biochar–mineral interactions, and uncertainties inherent to sequential extraction procedures. Fractionation results are operationally defined and may vary with extraction conditions, contributing to dispersion in measured values. Prior to statistical analysis, variables were examined for distributional properties and standardized to minimize scale effects. Interpretation of multivariate relationships considered these sources of variability and avoided reliance on single-factor correlations.

## 3 Results and discussion

### 3.1. Physicochemical properties of the initial soil and biochars

#### 3.1.1. Initial soil characteristics and metal contamination status

A detailed description of the initial soil properties provided the necessary background for interpreting Pb and Zn fractionation patterns and for understanding the subsequent response of these metals to biochar amendment. The key physicochemical parameters of the soil are presented in Table 2.

**Table 2:** Physicochemical properties of the studied soil^a^

Parameter	Unit	Value (mean ± SD)
Sand	%	66.71 ± 0.36
Silt	%	6.31 ± 0.23
Clay	%	26.98 ± 0.45
Textural class (USDA)	Sandy clay loam	
Reference classification (WRB)	Acrisols	
pH (H_2_O)	—	6.84 ± 0.01
Organic carbon (OC)	%	2.36 ± 0.05
Electrical conductivity (EC)	(µS cm^−1^)	52 ± 3
Cation exchange capacity (CEC)	cmol_c_ kg^−1^	10.5 ± 0.6
Pb (total)	mg kg^−1^	3920.3 ± 65.4
Zn (total)	mg kg^−1^	1789.9 ± 24.6
Cd (total)	mg kg^−1^	6.6 ± 0.2
Cu (total)	mg kg^−1^	21.1 ± 0.5

a Values are presented as mean ± standard deviation (*n* = 3). pH and EC were measured in a 1 : 5 soil–water suspension. CEC was determined using the ammonium acetate (1 M NH_4_OAc, pH 7.0 method).

The particle-size distribution revealed a predominance of sand (66.71 ± 0.36%), accompanied by lower proportions of silt (6.31 ± 0.23%) and a moderate clay content (26.98 ± 0.45%). On this basis, the soil was classified as sandy clay loam under the USDA textural system and as Acrisols according to the WRB classification. Soils with a high sand content are typically characterized by a relatively limited specific surface area, which restricts the retention of heavy metals *via* adsorption and ion-exchange mechanisms. Under such conditions, Pb and Zn are more likely to persist in mobile or weakly bound forms, increasing their susceptibility to dissolution and redistribution within the soil solution.

The initial soil exhibited a pH of 6.84 ± 0.01, corresponding to slightly acidic to near-neutral conditions. Under this pH range, Pb and Zn were expected to coexist in multiple chemical forms, including exchangeable species, carbonate-associated fractions, as well as forms bound to Fe/Mn oxides or organic matter. In metal-contaminated soils, such near-neutral pH conditions are generally insufficient to induce extensive precipitation of Pb and Zn as stable hydroxide or carbonate phases. As a consequence, a considerable proportion of these metals was likely to remain in mobile or weakly bound fractions.

The organic carbon (OC) content of the soil reached 2.36 ± 0.05%, indicating a moderate level of soil organic matter. Although soil organic matter can contribute to metal complexation, an OC content of this magnitude provided limited capacity for strong Pb and Zn immobilization through organic binding, particularly given the sandy texture of the soil. Electrical conductivity (EC) was relatively low, at 52 ± 3 µS cm^−1^, reflecting non-saline conditions and suggesting that overall ionic strength exerted only a minor influence on competitive metal adsorption. In contrast, the cation exchange capacity (CEC) was measured at 10.5 ± 0.6 cmol_c_ kg^−1^, which fell within the low to moderate range and indicated a restricted ability of the soil matrix to retain metal cations *via* ion-exchange processes.

Total concentrations of Pb and Zn were 3920.3 ± 65.4 mg kg^−1^ and 1789.9 ± 24.6 mg kg^−1^, respectively. These values exceeded national agricultural soil quality thresholds by a wide margin, considering that the corresponding regulatory limits for Pb and Zn are commonly set at 70 mg kg^−1^ and 200 mg kg^−1^. In addition to Pb and Zn, the soil also contained Cd (6.6 ± 0.2 mg kg^−1^) and Cu (21.1 ± 0.5 mg kg^−1^), with Cd concentrations surpassing the permitted guideline value of 5 mg kg^−1^. This metal assemblage reflected a multi-metal contamination scenario, characteristic of soils subjected to long-term impacts from mining activities or metallurgical operations. Among the detected metals, Pb and Zn dominated both in absolute concentration and environmental relevance, owing to their high toxicity and pronounced potential for bioaccumulation within soil–plant–human systems.

Taken together, the combination of a sand-rich texture, limited CEC, near-neutral pH, and exceptionally high total metal concentrations suggested that a substantial fraction of Pb and Zn was present in chemically labile or weakly bound forms. Under such conditions, assessments based solely on total metal contents were unlikely to provide an accurate representation of environmental risk or remediation performance.

For this reason, sequential chemical fractionation using the Tessier extraction scheme was applied to clarify the distribution of Pb and Zn among labile (F1–F2) and more stable fractions (F3–F5). This approach enabled a more refined evaluation of metal bioavailability and mobility, while also providing a robust framework for interpreting the mechanisms by which different biochars influenced Pb and Zn immobilization in subsequent sections of the study. Given that Pb and Zn concentrations greatly exceeded those of other metals such as Cd and Cu, the present investigation focused primarily on these two elements.

#### 3.1.2. Physicochemical properties of biochars derived from different biomass sources

The main physicochemical properties of biochars produced at 400 °C from sugarcane bagasse (SB), jackfruit seed (JB), and taro stem (TB) are summarized in Table 3. Although all biochars were generated under identical pyrolysis conditions, pronounced differences were observed in pH, specific surface area (BET), O/C atomic ratio, organic carbon (OC) content, and electrical conductivity (EC). These variations reflected the strong influence of the original biomass composition on the characteristics of the resulting biochars.

**Table 3:** Key physicochemical properties of the three biochars^a^

Biochar	pH	BET (m^2^ g^−1^)	O/C	OC (%)	EC (µS cm^−1^)
SB	9.15	50.1	0.13	85.95	847
JB	10.4	2.3	0.12	70.51	759
TB	10.08	32.8	0.21	44.56	4070

a SB, JB, and TB denote biochars produced at 400 °C from sugarcane bagasse, jackfruit seed, and taro stem, respectively. BET surface area was determined by N_2_ adsorption using the BET method. EC was measured in a 1 : 10 (w/v) biochar–water suspension.

All biochars exhibited alkaline pH values, ranging from 9.15 to 10.40. Among them, SB showed the lowest pH (9.15), whereas JB and TB displayed higher values of 10.40 and 10.08, respectively. These differences were closely related to the distinct biochemical compositions of the feedstocks. Sugarcane bagasse, which is rich in lignin, tended to retain a higher proportion of weakly acidic oxygen-containing functional groups (*e.g.*, –COOH and phenolic –OH) after pyrolysis at 400 °C, resulting in a comparatively lower degree of alkalinity. In contrast, the jackfruit seed biomass, dominated by cellulose and starch, underwent more extensive thermal decomposition, leading to a greater enrichment of alkaline mineral components in the solid phase and, consequently, a higher pH. For TB, the elevated alkalinity was likely associated with the substantial inorganic mineral content inherently present in *Colocasia* stems.

Clear differences were also evident in the BET surface area. SB exhibited the highest value (50.1 m^2^ g^−1^), followed by TB (32.8 m^2^ g^−1^), whereas JB showed a very limited surface area (2.3 m^2^ g^−1^). These contrasts highlighted the role of initial polymer structure in controlling pore development during pyrolysis. Lignin-rich biomass such as sugarcane bagasse favored the formation of a stable aromatic carbon framework and a well-developed porous structure. In comparison, jackfruit seed biomass, enriched in cellulose and starch, was more prone to softening and structural collapse during thermal treatment, which restricted pore formation and resulted in a low surface area. Despite its lower carbon content, TB still exhibited a moderately high surface area, likely due to the contribution of mineral phases in generating an inorganic porous structure.

The organic carbon content of the biochars followed the order SB (85.95%) > JB (70.51%) > TB (44.56%). This trend was consistent with the nature of the original feedstocks, as sugarcane bagasse contained a high proportion of lignocellulosic components that promoted the formation of stable carbon during pyrolysis, whereas *Colocasia* stem biomass contained a substantial fraction of mineral matter that diluted the carbon content of the resulting biochar. Differences in OC content were further reflected in the O/C atomic ratios. SB and JB showed relatively low O/C values (0.13 and 0.12), indicative of a higher degree of aromatization and comparatively hydrophobic surfaces. In contrast, TB exhibited the highest O/C ratio (0.21), suggesting a surface enriched with oxygen-containing functional groups and a greater potential for strong chemical interactions with metal ions.

Electrical conductivity varied widely among the biochars, ranging from 759 µS cm^−1^ for JB to 4070 µS cm^−1^ for TB. The exceptionally high EC of TB indicated a substantial presence of soluble ions and inorganic minerals capable of releasing alkaline and alkaline-earth cations into the soil solution. This characteristic was expected to contribute to soil pH elevation after amendment and to promote ion-exchange and precipitation processes, particularly for Pb and Zn. In contrast, the much lower EC values of SB and JB suggested that surface adsorption and complexation with organic functional groups were likely to play a more dominant role than mineral-driven mechanisms in their interactions with heavy metals.

Taken together, these results demonstrated that differences in biomass origin led to the formation of biochars with distinct physicochemical characteristics, even when produced under identical pyrolysis conditions. SB, representing lignin-rich biomass, combined high carbon content with a well-developed surface area, favoring physical adsorption and surface complexation mechanisms. JB, dominated by cellulose and starch, exhibited a poorly developed pore structure, implying that metal interactions were mainly governed by surface chemical reactions. In contrast, TB was characterized by high EC and a relatively elevated O/C ratio, highlighting the importance of mineral-associated mechanisms such as ion exchange, precipitation, and chemical bonding with oxygen-containing functional groups.

These systematic differences provided a critical basis for interpreting the contrasting Pb and Zn immobilization behaviors observed among the biochars in subsequent sections.

### 3.2. Surface morphology and functional characteristics of biochars

#### 3.2.1. SEM-EDS analysis

**Fig. 3:**
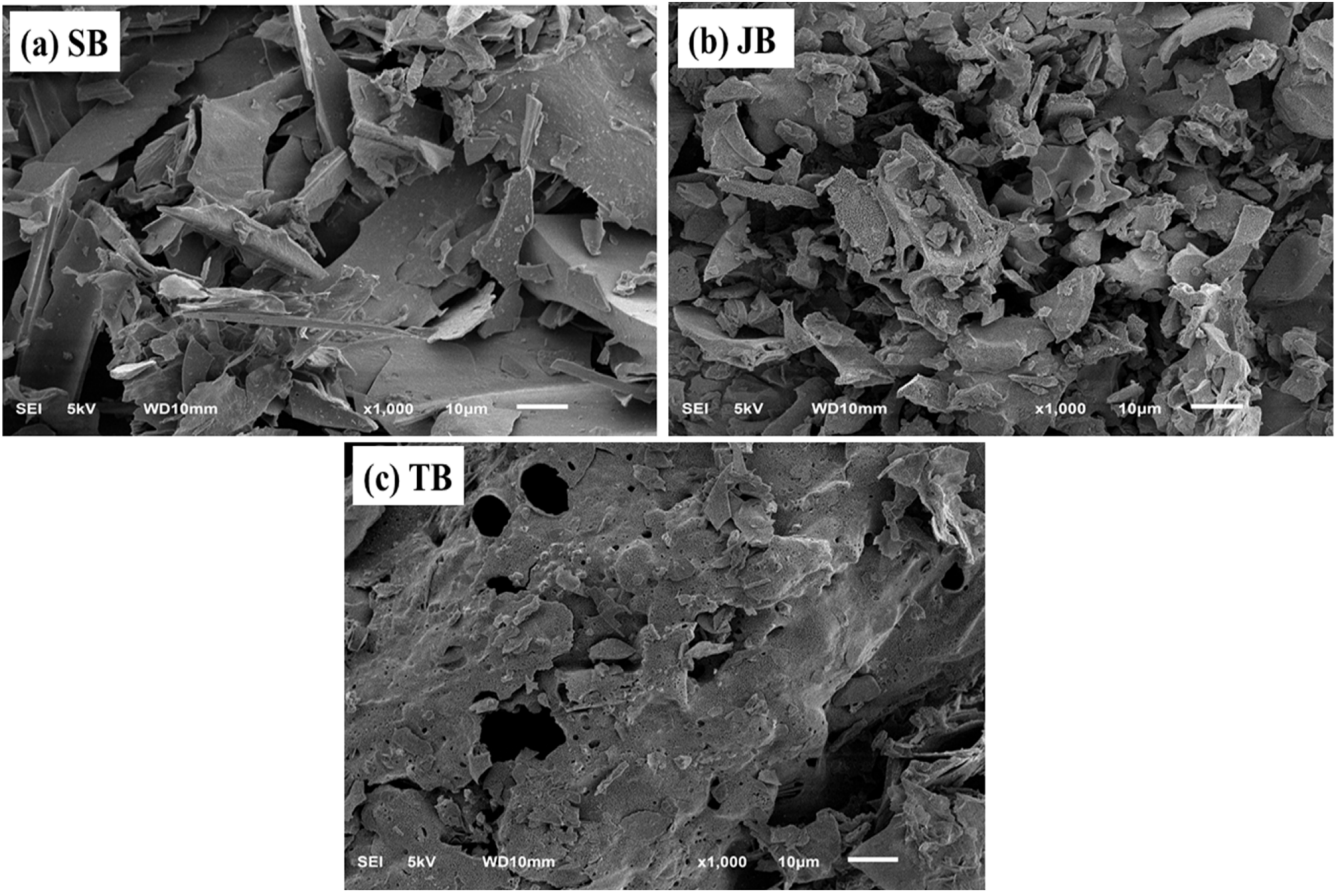
SEM images of biochars produced at 400 °C: (a) SB, (b) JB, and (c) TB. Arrows highlight representative pore structures and surface features. Scale bars correspond to 10 µm.

Scanning electron microscopy (SEM) combined with energy-dispersive X-ray spectroscopy (EDS) was employed to examine differences in surface morphology, porosity, and inorganic elemental composition among the SB, JB, and TB biochars. These observations provided a visual basis for interpreting the immobilization mechanisms of Pb and Zn in contaminated soils. The SEM-EDS results are presented in Fig. 3.

SEM micrographs revealed pronounced contrasts in surface structure among the biochars derived from different biomass sources. Sugarcane bagasse biochar (SB) exhibited a relatively well-developed surface architecture, characterized by a network of pores with fairly uniform distribution, including slit-shaped and irregular pores (Fig. 3a). This morphology was consistent with the highest BET surface area measured for SB and reflected the influence of the lignin-rich nature of sugarcane bagasse, which favored the formation of a stable aromatic carbon framework and preservation of porosity during pyrolysis. Such structural features were conducive to surface adsorption and ion-exchange processes, particularly for mobile Pb and Zn species in soil.

In contrast, jackfruit seed biochar (JB) displayed a comparatively compact surface with limited pore development and a less hierarchical structure (Fig. 3b). SEM images showed that JB particles tended to agglomerate, with only a small number of visible pores, in agreement with its very low BET surface area. This morphology was attributed to the cellulose- and starch-rich composition of the jackfruit seed biomass, which underwent extensive thermal degradation, leading to pore collapse during pyrolysis at 400 °C. As a consequence, Pb and Zn immobilization by JB was more likely governed by chemical interactions with surface functional groups (*e.g.*, –OH and –COOH) and by indirect pH-mediated effects, rather than by physical adsorption.

For taro stem biochar (TB), SEM images revealed a distinct surface structure consisting of medium to large pores interspersed with irregularly distributed inorganic phases on the carbon matrix (Fig. 3c). The presence of protruding mineral particles on the biochar surface represented a characteristic feature of TB and reflected the high ash and mineral content of the original biomass. This composite structure provided conditions that may facilitate precipitation and co-precipitation processes involving Pb and Zn with mineral phases.

**Fig. 4:**
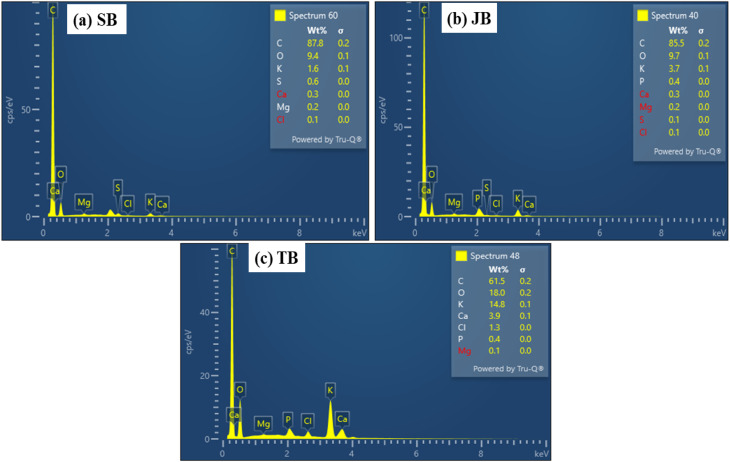
EDS spectra of biochars: (a) SB, (b) JB, and (c) TB. Major elemental peaks (Ca, Mg, K, Si, and P) are indicated to illustrate mineral composition differences among biochars.

EDS analysis supported the morphological observations by confirming the presence of various mineral elements, including Ca, Mg, K, Si, and P, on the surfaces of all biochars (Fig. 4a–c), with particularly high abundances detected for TB (Fig. 4c).

The enrichment of Ca and Mg in TB indicates that mineral-associated immobilization pathways may play an important role. Under such conditions, the formation of Pb- or Zn-bearing carbonate and phosphate phases (*e.g.*, PbCO_3_, Pb_3_(PO_4_)_2_, ZnCO_3_) can be considered as plausible pathways facilitating the transfer of Pb and Zn from mobile fractions (F1) to more stable fractions (F2–F5). However, these phase-specific products are not directly confirmed by SEM-EDS and should therefore be interpreted as hypothetical rather than definitive. It should be noted that SEM-EDS provides elemental rather than phase-specific information, and thus cannot resolve the exact mineral forms responsible for immobilization. SB also exhibited appreciable levels of alkaline and alkaline-earth elements, which may contribute to Pb and Zn stabilization through ion-exchange processes and pH elevation following amendment, thereby reducing metal solubility (Fig. 4a).

The combined SEM-EDS observations demonstrated that biomass origin controlled not only the physical structure of the biochars but also the chemical nature of their surfaces, with direct implications for heavy metal immobilization mechanisms. Taro stem biochar, characterized by a moderately developed porous structure and high mineral content, likely promotes Pb and Zn immobilization *via* mineral-associated pathways. These may include precipitation processes, although they cannot be directly confirmed by SEM-EDS. In comparison, SB primarily promoted adsorption and ion-exchange processes, whereas JB relied largely on surface functional group interactions and pH-related effects.

#### 3.2.2. FT-IR analysis and surface functional group distribution

**Fig. 5:**
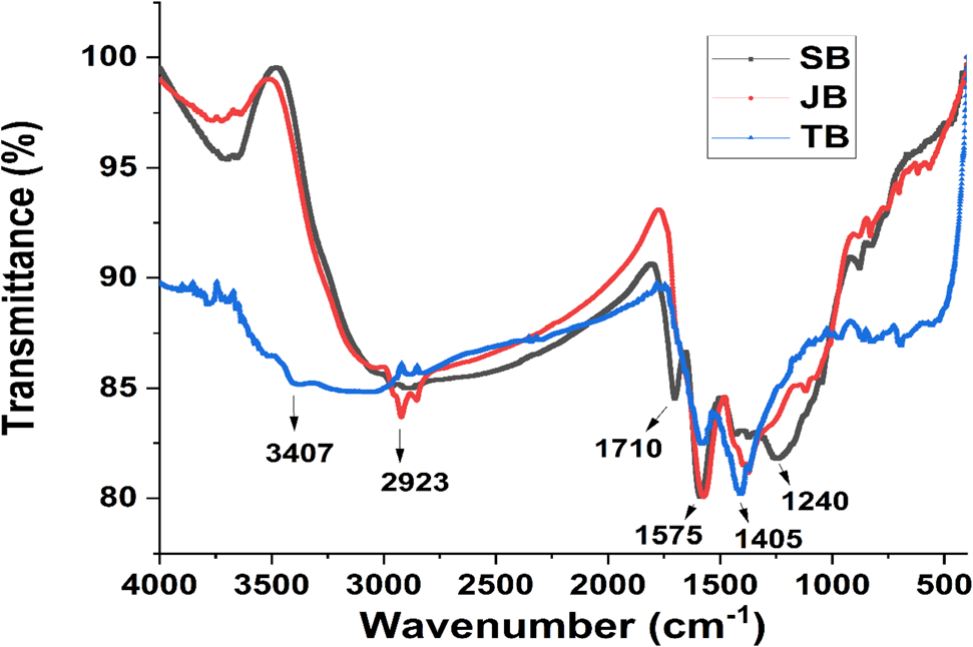
FT-IR spectra of the three biochars: sugarcane bagasse (SB), jackfruit seed biochar (JB) and taro stem biochar (TB).

Fourier-transform infrared (FT-IR) spectroscopy was applied to identify the dominant surface functional groups of the SB, JB, and TB biochars and to clarify the role of chemical interactions in Pb and Zn immobilization. The FT-IR spectra of the three biochars (Fig. 5) showed the coexistence of oxygen-containing functional groups, aromatic carbon structures, and vibrations associated with inorganic mineral phases. Noticeable differences in band intensity and distribution were observed among the materials, reflecting variations in biomass origin and surface chemistry.

A broad absorption band in the range of 3200–3600 cm^−1^ was present in all spectra and was attributed to O–H stretching vibrations of hydroxyl groups associated with alcohols, phenols, and adsorbed water on biochar surfaces. The intensity of this band was markedly higher for SB and TB than for JB, indicating a higher surface density of hydroxyl groups. These –OH groups may act as potential reactive sites for Pb^2+^ binding, although direct evidence of complex formation was not obtained. Previous studies have reported a strong affinity of Pb for phenolic –OH and carboxyl groups on biochar surfaces, leading to the formation of stable inner-sphere complexes.

Distinct absorption bands in the regions of 1700–1725 cm^−1^ and 1580–1620 cm^−1^ were assigned to C=O stretching vibrations of carboxyl groups (–COOH) and to aromatic C=C stretching, respectively. In the SB spectrum, these bands were sharper and more intense than those observed for JB and TB, indicating a higher abundance of aromatic carbon and carboxyl functional groups. This observation was consistent with the lignin-rich nature of sugarcane bagasse. Carboxyl groups play a critical role in chelation with Pb^2+^, and Pb generally forms more stable complexes than Zn due to its larger ionic radius and higher polarizability. By contrast, the FT-IR spectrum of JB showed relatively weak signals associated with –COOH and aromatic C=C groups, suggesting a lower degree of carbon condensation and a limited density of oxygen-containing functional groups. This implied that metal immobilization by JB relied less on strong surface complexation and more on electrostatic attraction and ion-exchange processes. Such mechanisms are particularly relevant for Zn^2+^, which tends to interact more weakly with organic functional groups than Pb^2+^. Consistent with this interpretation, recent studies have reported that Zn is commonly retained on biochar surfaces *via* outer-sphere complexation or exchange with alkali and alkaline-earth cations (*e.g.*, Na^+^, K^+^, Ca^2+^), rather than through the formation of stable inner-sphere complexes.

In the case of TB, in addition to the O–H and aromatic C=C bands, distinct absorptions were observed in the 1000–1100 cm^−1^ region. These bands were commonly attributed to Si–O–Si or P–O vibrations associated with silicate and phosphate mineral phases inherited from the taro stem biomass. The presence of such inorganic functional groups suggested that precipitation or co-precipitation processes involving Pb and Zn as metal phosphates or carbonates may occur under these conditions; however, direct evidence for specific mineral phases is not provided by FT-IR analysis. This pathway is considered especially effective for Pb immobilization due to the extremely low solubility of Pb-phosphate phases, whereas Zn typically forms less stable precipitates.

Overall, the FT-IR results indicated a clear divergence in immobilization behavior between Pb and Zn. Pb and Zn exhibited distinct response patterns under the same experimental conditions, with Pb showing stronger associations with –COOH and –OH functional groups, particularly in SB and TB, while Zn was more frequently associated with less specific processes such as electrostatic adsorption and ion exchange, especially in materials with lower densities of organic functional groups, such as JB. These differences reflected the intrinsic chemical properties of the two metals and explained why a single biochar type exhibited contrasting immobilization efficiencies for Pb and Zn.

The FT-IR analysis provided spectroscopic evidence supporting the chemical mechanisms governing Pb and Zn immobilization and established a basis for metal-specific interpretation in subsequent sections. The identified functional group characteristics also served as key explanatory variables for the PCA and feature-importance analyses in the machine-learning models (RF and XGBoost), thereby linking spectroscopic information with the redistribution of Pb and Zn among chemical fractions in soil.

### 3.3. Effects of biochar type and application rate on soil properties after incubation

#### 3.3.1. Changes in soil pH following biochar amendment

Changes in soil pH, organic carbon (OC), and electrical conductivity (EC) were evaluated for the control soil and for soils amended with different biochars at varying application rates. As summarized in Table 4, biochar addition resulted in a clear increase in soil pH compared with the unamended control (BS), with statistically significant differences confirmed by ANOVA (*p* < 0.05). The magnitude of this increase varied systematically with both biochar type and application rate (*p* < 0.05; see Table S2 in the SI).

**Table 4:** Effects of biochar amendment rate on soil pH, organic carbon (OC), and electrical conductivity (EC) after 1 month incubation. Different letters within the same column indicate significant differences (Tukey HSD, *p* < 0.05)^a^

Sample	Rate (%)	pH	OC (%)	EC (µS cm^−1^)	ΔpH	ΔOC	ΔEC
BS	0	6.84 ± 0.08^d^	2.36 ± 0.05^d^	52 ± 3^e^	0	0	0
SB3	3	7.02 ± 0.05^c^	2.92 ± 0.07^b^	76 ± 4^d^	0.18	0.56	24
SB5	5	7.16 ± 0.06^b^	3.28 ± 0.08^a^	98 ± 5^c^	0.32	0.92	46
SB10	10	7.34 ± 0.07^a^	3.82 ± 0.10^a^	138 ± 7^b^	0.5	1.46	86
JB3	3	7.08 ± 0.05^c^	2.78 ± 0.06^bc^	74 ± 4^d^	0.24	0.42	22
JB5	5	7.20 ± 0.06^b^	3.02 ± 0.07^b^	96 ± 5^c^	0.36	0.66	44
JB10	10	7.36 ± 0.07^a^	3.46 ± 0.08^ab^	132 ± 7^b^	0.52	1.1	80
TB3	3	7.15 ± 0.06^b^	2.60 ± 0.06^c^	118 ± 6^b^	0.31	0.24	66
TB5	5	7.25 ± 0.03^b^	2.70 ± 0.05^c^	165 ± 8^a^	0.41	0.34	113
TB10	10	7.55 ± 0.08^a^	3.5 ± 0.08^b^	240 ± 12^a^	0.71	0.69	188

a Values are means ± standard deviations (*n* = 3). Different letters indicate significant differences at *p* < 0.05 according to Tukey's HSD test. BS represents Pb/Zn contaminated soil without biochar amendment (control soil or blank soil). SB3-SB10, JB3-JB10, and TB3-TB10 indicate contaminated soils amended with 3%, 5%, and 10% (w/w) of the sugarcane bagasse, jackfruit seed, and taro stem biochars, respectively. ΔpH, ΔOC, and ΔEC represent the changes in soil pH, organic carbon content, and electrical conductivity, respectively, relative to the control soil (BS).

The initial pH of the control soil was 6.84 ± 0.08, corresponding to slightly acidic to near-neutral conditions. At this pH range, Pb and Zn were expected to remain partially in labile chemical forms (F1–F2), consistent with their relatively high mobility in contaminated soils.

Following biochar incubation, soil pH increased progressively with increasing amendment rate from 3% to 10% (w/w). For sugarcane bagasse biochar (SB), soil pH increased from 7.02 at 3% to 7.34 at 10%. A comparable trend was observed for jackfruit seed biochar (JB), with pH rising from 7.08 to 7.36 across the same application range. In contrast, taro stem biochar (TB) produced the most pronounced pH elevation, particularly at the 10% rate, where soil pH reached 7.55 ± 0.08. This value was significantly higher than those observed for SB and JB at the same dosage (*p* < 0.05; ANOVA results provided in Table S2 in the SI).

The observed increase in soil pH after biochar amendment was mainly attributed to the high ash content and the release of basic cations (Ca^2+^, Mg^2+^, K^+^, and Na^+^) from the biochars into the soil solution. Among the three materials, TB exhibited the highest EC and mineral content (Table 4), explaining its stronger acid-neutralizing capacity and greater ability to elevate soil pH relative to SB and JB. Similar pH-adjustment effects have been widely reported for mineral-rich biochars, which are known to reduce the mobility of potentially toxic elements such as Pb and Zn through chemical buffering processes.

The increase in soil pH played a critical role in controlling Pb and Zn immobilization. Higher pH conditions reduced metal solubility and may promote processes associated with carbonate-bound fractions, as reflected by the increase in F2. However, this should not be interpreted as direct evidence of specific carbonate precipitation (*e.g.*, PbCO_3_), as the Tessier extraction defines operational fractions rather than discrete mineral phases. As a result, pH changes acted as a key environmental driver governing the redistribution of Pb and Zn from labile fractions toward more stable chemical forms, a process examined in detail in Section 3.4.

#### 3.3.2. Changes in soil organic carbon (OC) after biochar incubation

Alongside pH variation, changes in soil organic carbon (OC) following biochar incubation were quantified and are summarized in Table 4. Relative to the control soil, OC increased markedly after biochar addition, reflecting the direct input of recalcitrant carbon from the amended materials. The OC content of the control soil was 2.36 ± 0.05%, whereas soils amended with biochar exhibited a dose–dependent increase for all three materials (*p* < 0.05).

After one month of incubation, sugarcane bagasse biochar (SB) produced the largest OC enrichment, reaching 3.82 ± 0.10% at the 10% application rate, corresponding to an increase of approximately 62% compared with the control. Jackfruit seed biochar (JB) also increased OC, though to a lesser extent (3.46 ± 0.08% at 10%), while taro stem biochar (TB) resulted in the lowest OC values among the amended treatments (3.05 ± 0.08% at 10%). This pattern was consistent with the lower intrinsic OC content of TB relative to SB and JB (Table 3).

The increase in soil OC extended beyond a simple mass balance effect and carried mechanistic implications for metal immobilization. Organic carbon, particularly aromatic carbon domains and oxygen-containing surface functional groups associated with biochar, provided effective binding sites for Pb through stable complexation and contributed adsorption sites for Zn. Recent studies have identified OC as one of the most influential variables explaining reductions in labile Pb fractions following biochar amendment, supporting the relevance of OC enrichment to Pb stabilization in contaminated soils.

#### 3.3.3. Changes in electrical conductivity (EC) and the role of background ions

Like pH and OC, soil electrical conductivity (EC) increased substantially after biochar incubation, with the magnitude of change strongly dependent on both biochar type and application rate. While SB and JB induced moderate EC increases (maximum values of approximately 138 and 132 µS cm^−1^ at the 10% rate, respectively), TB produced a much stronger response, reaching 240 ± 12 µS cm^−1^ at the same dosage. This value was nearly five times higher than that measured in the control soil.

Elevated EC values reflected the release of soluble ions from biochar, including base cations and carbonate/bicarbonate anions, into the soil solution. These ions influenced metal behavior by competing with Pb^2+^ and Zn^2+^ at exchange sites and by facilitating ion-exchange reactions and secondary mineral precipitation, particularly for Pb. At the same time, excessive ionic strength can enhance the mobility of certain metals through background electrolyte effects. For this reason, EC changes were evaluated together with metal fraction redistribution to capture both stabilizing and potentially mobilizing influences of dissolved ions.

#### 3.3.4. Mechanistic implications and linkage to Pb/Zn redistribution

The results demonstrated that biochar amendment substantially modified the soil chemical environment through concurrent increases in pH, OC, and EC, with distinct magnitudes among biochar types and application rates. These changes created conditions favorable for Pb and Zn immobilization *via* pH-dependent processes, complexation with organic carbon, and mineral–ion interactions.

It should be emphasized that the present dataset, derived from a single-soil incubation system with treatment-level predictors, does not allow mechanistic resolution of competitive adsorption processes between Pb and Zn. Observed differences therefore reflect co-existing responses under shared conditions rather than direct competition for binding sites. Accordingly, the following discussion focuses on comparative trends rather than mechanistic competition.

Among the three materials, TB showed the strongest capacity to modify pH and EC, highlighting the importance of precipitation and co-precipitation pathways. In contrast, SB was particularly effective in enhancing soil OC and surface functional group availability, favoring Pb complexation. These systematic differences were associated with the redistribution of Pb and Zn among Tessier fractions (F1–F5), reflected in shifts from labile to more stable forms under altered soil chemical conditions.

### 3.4. Redistribution of Pb and Zn chemical forms following biochar amendment

#### 3.4.1. Changes in Pb chemical fractionation

**Fig. 6:**
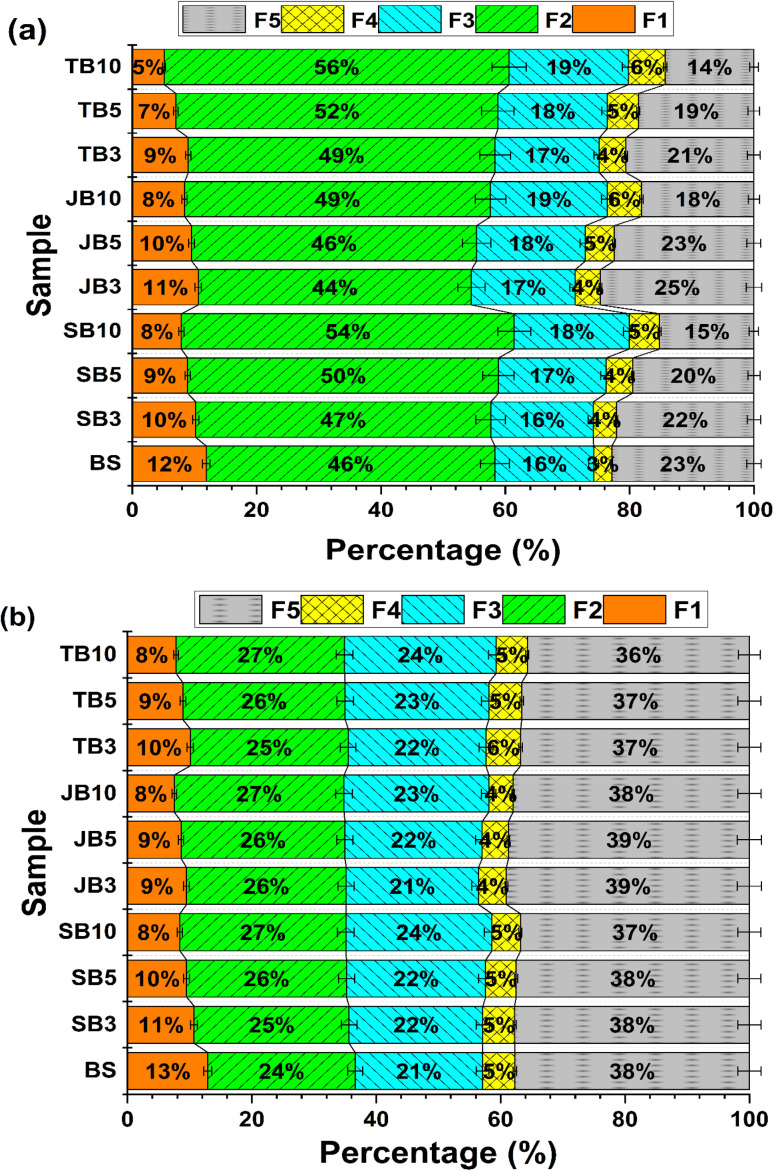
Distribution of Tessier fractions of Pb (a) and Zn (b) in soil after incubation (incubation time: 30 days; *n* = 3; fractions expressed as % of total). Data are presented as mean ± standard deviation (*n* = 3). Different letters indicate statistically significant differences at *p* < 0.05.

Lead concentrations (mg kg^−1^) in soils before and after biochar incubation at different application rates are summarized in Table 5, while the percentage distribution of Pb among chemical fractions is presented in Table S3 (SI) and Fig. 6. A two-way ANOVA was performed to evaluate the effects of biochar type and application rate on the distribution of Pb fractions (Table S4, SI). Among all fractionation changes, the most pronounced and consistent response to biochar amendment was the substantial reduction in the exchangeable Pb fraction (F1), which represents the most mobile and bioavailable pool. This decrease was strongly dose–dependent and statistically significant across all biochar types (*p* < 0.05), highlighting F1 depletion as the central immobilization pathway in the present study.

**Table 5:** Distribution of Pb concentrations among Tessier sequential extraction fractions (F1–F5, mg kg^−1^) as affected by biochar type and application rate (mean ± SD, *n* = 3)^a^

Sample	Rate (%)	F1	F2	F3	F4	F5
BS	0	466.5 ± 32.5^a^	1822.9 ± 68.4^b^	622.6 ± 41.7^c^	113.4 ± 9.8^d^	896.5 ± 37.2^a^
SB3	3	387.5 ± 30.2^b^	1810.4 ± 63.7^b^	630.1 ± 39.4^bc^	144.3 ± 11.1^c^	842.7 ± 35.6^b^
SB5	5	331.8 ± 25.7^c^	1862.9 ± 60.4^ab^	644.5 ± 36.8^b^	159.6 ± 12.3^bc^	727.2 ± 30.8^c^
SB10	10	280.6 ± 20.1^d^	1909.8 ± 56.9^a^	659.7 ± 33.5^ab^	174.6 ± 13.1^b^	541.4 ± 22.7^d^
JB3	3	402.1 ± 31.6^d^	1676.3 ± 59.2^c^	634.9 ± 38.1^bc^	155.8 ± 11.8^bc^	939.1 ± 38.9^a^
JB5	5	354.6 ± 26.9^c^	1712.8 ± 56.4^c^	651.6 ± 35.7^b^	171.9 ± 12.6^b^	840.6 ± 33.4^b^
JB10	10	299.4 ± 22.3^d^	1756.2 ± 53.1^bc^	670.1 ± 32.4^ab^	194.7 ± 13.9^ab^	645.5 ± 26.5^c^
TB3	3	343.8 ± 27.4^c^	1882.7 ± 64.8^ab^	639.4 ± 37.9^bc^	162.6 ± 12.1^bc^	783.5 ± 32.6^b^
TB5	5	260.5 ± 21.8^d^	1932.4 ± 61.5^a^	657.8 ± 35.2^ab^	187.9 ± 13.4^ab^	692.4 ± 28.7^c^
TB10	10	182.6 ± 16.5^e^	1979.8 ± 58.3^a^	684.9 ± 31.6^a^	211.6 ± 14.7^a^	509.2 ± 21.9^d^

a F1, exchangeable; F2, carbonate-bound; F3, Fe/Mn oxide-bound; F4, organic matter-bound; F5, residual fraction. Values represent mean concentrations (*n* = 3). Control (BS) refers to soil without biochar amendment. SB, JB, and TB denote sugarcane bagasse, jackfruit seed, and taro stem biochars produced at 400 °C, respectively. Different lowercase letters within each column indicate significant differences (2-way ANOVA (biochar type × dose) + Tukey HSD, *p* < 0.05).

In the unamended soil, F1_Pb accounted for 11.9% of total Pb, indicating a considerable proportion of labile Pb. Following biochar addition, F1_Pb decreased markedly with increasing application rate, reaching 7.9% (SB10), 8.4% (JB10), and as low as 5.1% (TB10). In absolute terms, F1_Pb declined from 464.6 mg kg^−1^ in the control to 182.6 mg kg^−1^ in TB10, corresponding to a reduction of approximately 61%.

In the unamended control soil (BS), Pb was distributed among the five Tessier fractions in the following order: F2 > F5 > F3 > F1 > F4. Carbonate-bound Pb (F2) dominated the speciation, accounting for 46.5% of total Pb, followed by the residual fraction (F5, 22.9%) and the Fe/Mn oxide-bound fraction (F3, 15.9%). Notably, the exchangeable fraction (F1) still represented 11.9% of total Pb (Table S3; Fig. 5), indicating relatively high mobility and potential bioavailability in the control soil.

After 30 days of incubation, biochar amendment markedly altered Pb partitioning, with a pronounced reduction in the exchangeable fraction (F1). For all biochar types, F1_Pb decreased progressively with increasing application rate. Specifically, F1_Pb declined from 464.6 mg kg^−1^ in the control to 280.6 mg kg^−1^ for SB10, 299.4 mg kg^−1^ for JB10, and reached the lowest value of 182.6 mg kg^−1^ for TB10 (ANOVA, *p* < 0.05). In relative terms, the percentage contribution of F1 decreased from 11.9% to 7.9% (SB10), 8.4% (JB10), and 5.1% (TB10) (Fig. 6; Table S5 (SI)).

This pronounced reduction in F1_Pb indicates a clear shift from labile to less mobile forms, driven primarily by pH-induced changes and enhanced surface interactions following biochar amendment. Increased pH reduced Pb solubility and altered surface charge characteristics, thereby decreasing the stability of exchangeable Pb and facilitating its redistribution into less labile fractions. As pH increased, negative surface charges on clay minerals and organic matter became more pronounced, while Pb^2+^ was progressively displaced from exchange sites and transferred into more stable forms through precipitation reactions and surface complexation. Similar patterns have been widely reported, with soil pH elevation and biochar ash content identified as dominant factors controlling the reduction of exchangeable Pb. In contrast, the carbonate-bound fraction (F2) showed a consistent, albeit moderate, increase with rising biochar application rate, particularly at 10%. The proportion of Pb associated with F2 increased from 46.5% in the control soil to 53.6% (SB10), 49.2% (JB10), and 55.5% (TB10). In parallel, a moderate increase in the carbonate-bound fraction (F2) was observed, suggesting a partial redistribution of Pb into less labile pools. However, this change should be interpreted cautiously, as F2 represents an operationally defined fraction and does not directly indicate the formation of specific carbonate minerals. Such carbonate-driven stabilization pathways have been consistently documented for alkaline and mineral-rich biochars and were especially pronounced for TB in the present study.

In addition to the marked decline in labile Pb forms, biochar amendment promoted the redistribution of Pb toward more stable fractions, particularly the Fe/Mn oxide-bound fraction (F3) and the organic matter-bound fraction (F4). The concentration of Pb associated with F3 increased consistently with increasing biochar application rate for all three biochars. For example, Pb–F3 rose from 622.6 mg kg^−1^ in the control soil to 684.9 mg kg^−1^ in the TB10 treatment, corresponding to an increase in relative contribution from 15.9% to 19.2%. This trend reflected the strong affinity of Pb for Fe/Mn oxide surfaces and suggested that biochar amendment facilitated the formation of stable oxide–organic assemblages capable of retaining Pb over longer timescales.

A pronounced increase was also observed for the organic matter-bound fraction (F4) following biochar incubation. The percentage of Pb–F4 increased from 2.9% in the control soil to 4.9% (SB10), 5.5% (JB10), and reached 5.9% in TB10. This shift indicated enhanced inner-sphere complexation of Pb with oxygen-rich functional groups on biochar surfaces, including carboxyl, phenolic hydroxyl, and aromatic structures. Compared with Zn, Pb exhibited a greater tendency to form strong chemical bonds with these functional groups, explaining its preferential stabilization within the F4 fraction after biochar amendment.

In contrast, the residual fraction (F5) showed a slight decrease with increasing biochar application rate, particularly for SB and TB treatments, where the contribution of F5 declined from 22.9% in the control soil to 14.3% in TB10. This decrease did not imply remobilization of Pb, but rather reflected a redistribution of Pb from original mineral phases into newly formed, biochar-associated stable phases. Within the operational framework of the Tessier sequential extraction, such phases were classified as F3 or F4, a phenomenon that has been widely recognized as an inherent limitation of sequential extraction procedures. It is important to recognize that the Tessier sequential extraction scheme is operationally defined and may not uniquely distinguish between specific binding mechanisms. Redistribution among fractions should therefore be interpreted cautiously, as fraction boundaries may overlap and transformation pathways cannot be directly resolved.

##### 3.4.1.1 Comparison of Pb immobilization efficiency among biochars

Among the three biochars, taro stem-derived biochar (TB) exhibited the highest Pb immobilization efficiency, followed by sugarcane bagasse biochar (SB), while jackfruit seed biochar (JB) showed the lowest performance. At the 10% application rate, TB resulted in the lowest proportion of F1_Pb (5.1%) and the highest combined contribution of stable fractions (F3 + F4, 25.1%). This superior performance was consistent with the high ash content, strong alkalinity, and greater release of alkaline and alkaline-earth cations from TB, which jointly promoted precipitation reactions, specific adsorption, and surface complexation processes.

Overall, the fractionation results clearly demonstrate that the dominant effect of biochar amendment was the substantial depletion of the exchangeable Pb fraction (F1), accompanied by a redistribution toward less labile pools. This shift provides robust and quantitative evidence for reduced Pb mobility and bioavailability, and represents the central outcome of the present study.

#### 3.4.2. Redistribution of Zn chemical fractions following biochar amendment

The distribution of Zn chemical fractions was summarized in Tables 6 and S5 (SI), and Fig. 5b. A two-way ANOVA was performed to evaluate the effects of biochar type and application rate on the distribution of Zn fractions (Table S6, SI). In the unamended control soil (BS), Zn occurred predominantly in the residual fraction (F5, 37.7%), followed by the carbonate-bound fraction (F2, 23.7%), the Fe/Mn oxide-bound fraction (F3, 20.5%), and the exchangeable fraction (F1, 12.9%), whereas the organic matter-bound fraction (F4) accounted for the smallest proportion (∼5%). This pattern indicated that, despite a substantial pool of Zn associated with stable mineral phases, a non-negligible fraction remained potentially mobile and bioavailable.

**Table 6:** Distribution of Zn concentrations among Tessier sequential extraction fractions (F1–F5, mg kg^−1^) as affected by biochar type and application rate (mean ± SD, *n* = 3)^a^

Sample	Rate (%)	F1 (mg kg^−1^)	F2 (mg kg^−1^)	F3 (mg kg^−1^)	F4 (mg kg^−1^)	F5 (mg kg^−1^)
BS	0	230.5 ± 18.6^a^	424.6 ± 21.3^c^	366.7 ± 19.4^c^	93.1 ± 5.8^a^	675.1 ± 17.9^a^
SB3	3	185.6 ± 15.9^b^	432.7 ± 20.8^bc^	374.5 ± 17.3^bc^	89.3 ± 5.4^ab^	655.8 ± 16.9^ab^
SB5	5	162.3 ± 14.3^c^	438.1 ± 21.2^bc^	382.9 ± 18.2^bc^	83.4 ± 5.1^bc^	641.2 ± 15.8^bc^
SB10	10	139.8 ± 13.1^d^	444.5 ± 22.1^ab^	391.6 ± 18.7^ab^	76.9 ± 4.8^cd^	612.4 ± 15.1^cd^
JB3	3	158.4 ± 14.6^c^	429.6 ± 20.4^c^	356.1 ± 16.9^c^	74.8 ± 4.7^d^	653.2 ± 16.4^ab^
JB5	5	142.6 ± 13.4^d^	435.2 ± 20.9^bc^	364.7 ± 17.2^bc^	69.5 ± 4.5^de^	640.1 ± 15.9^bc^
JB10	10	121.3 ± 11.9^e^	441.8 ± 21.4^ab^	375.9 ± 17.8^bc^	62.4 ± 4.2^e^	614.6 ± 15.3^cd^
TB3	3	176.9 ± 15.6^b^	446.3 ± 22.3^ab^	391.4 ± 18.6^ab^	98.5 ± 6.1^a^	646.8 ± 16.2^ab^
TB5	5	154.2 ± 14.1^c^	451.7 ± 22.8^a^	399.6 ± 19.1^ab^	91.2 ± 5.6^ab^	633.1 ± 15.6^bc^
TB10	10	131.6 ± 12.8^de^	458.4 ± 23.4^a^	412.8 ± 19.7^a^	83.7 ± 5.7^bc^	602.9 ± 14.9^d^

a F1, exchangeable; F2, carbonate-bound; F3, Fe/Mn oxide-bound; F4, organic matter-bound; F5, residual fraction. Values represent mean concentrations (*n* = 3). BS refers to soil without biochar amendment. Different lowercase letters within each column indicate significant differences (2-way ANOVA (biochar type × dose) + Tukey HSD, *p* < 0.05).

Consistent with the behavior observed for Pb, Zn exhibited a decline in the exchangeable fraction (F1) with increasing biochar application rate. The concentration of F1_Zn decreased from 230.5 mg kg^−1^ in the control soil to 139.8 mg kg^−1^ (SB10), 121.3 mg kg^−1^ (JB10), and 131.6 mg kg^−1^ (TB10). In relative terms, the contribution of F1_Zn declined from 12.9% to 8.4%, 7.5%, and 7.8% for SB10, JB10, and TB10, respectively (Table S6). However, the magnitude of this reduction remained markedly lower than that observed for Pb, suggesting that Zn was less efficiently immobilized at exchangeable sites in biochar-amended soils, a trend frequently reported in studies employing the Tessier sequential extraction scheme.

##### 3.4.2.1 Role of pH regulation and ion-exchange-dominated mechanisms for Zn

Unlike Pb, which showed a pronounced redistribution toward Fe/Mn oxide-bound (F3) and organic matter-bound (F4) fractions, Zn was mainly redistributed toward carbonate-bound (F2) and Fe/Mn oxide-bound (F3) forms with increasing biochar dose. The proportion of F2 (Zn) increased from 23.7% in the control soil to approximately 26.7–27.3% at the 10% biochar treatments, while F3 increased from 20.5% to 23.5–24.4% (Table S6; Fig. 5b). This pattern reflected the strong dependence of Zn behavior on soil pH and ion-exchange processes. Elevated pH reduced Zn solubility and promoted association with carbonate phases and mineral oxides, yet was insufficient to drive extensive formation of stable inner-sphere complexes comparable to those observed for Pb. Consequently, Zn retention was dominated by exchange reactions with alkaline and alkaline-earth cations and by adsorption onto mineral surfaces, which are inherently less stable than organic complexation mechanisms.

##### 3.4.2.2 Competitive adsorption between Zn and Pb

Competitive adsorption between Zn and Pb also influenced Zn immobilization. Due to its larger ionic radius and higher polarizability, Pb^2+^ preferentially occupied high-affinity functional sites on biochar surfaces, such as carboxyl and phenolic hydroxyl groups. This preferential binding constrained the access of Zn^2+^ to these sites, forcing Zn to associate more frequently with weaker, outer-sphere adsorption environments. This competition was reflected in the organic matter-bound fraction (F4), where the relative contribution of Zn slightly declined from ∼5.2% in the control soil to approximately 4–5% in biochar-amended treatments, in contrast to the clear enrichment of Pb within the same fraction.

##### 3.4.2.3 Residual fraction (F5) and long-term stability of Zn

The residual fraction (F5) remained the dominant Zn pool across all treatments, showing only a minor decrease with increasing biochar dose while maintaining high contributions (36–38%). This observation indicated that most Zn remained associated with stable mineral phases inherent to the soil matrix. Overall, although biochar amendment reduced the labile Zn pool and promoted partial redistribution toward less mobile fractions (F2 and F3), Zn consistently exhibited a lower degree of immobilization than Pb under comparable conditions, a characteristic widely documented in chemical fractionation studies.

##### 3.4.2.4 Comparison of Zn immobilization among biochars

Among the three biochars, no pronounced differences were observed in terms of F1_Zn reduction or enrichment of stable Zn fractions. Only at the highest application rate (10%) did TB show a slight tendency toward increased F3_Zn, consistent with the presence of abundant mineral phases capable of providing additional inorganic sorption sites. Nevertheless, the overall response of Zn remained weaker than that of Pb, emphasizing that metal-specific properties exerted a stronger control on immobilization mechanisms than the intrinsic characteristics of the amendment alone.

##### 3.4.2.5 Direct comparison of Pb and Zn immobilization mechanisms

A direct comparison of Pb and Zn fractionation under identical experimental conditions highlighted clear mechanistic differences between the two metals. Although both Pb and Zn showed reductions in the exchangeable fraction (F1) following biochar amendment, Pb responded more strongly. For Pb, the F1 concentration decreased from 464.6 mg kg^−1^ (11.9%) in the control soil to 182.6 mg kg^−1^ (5.1%) in TB10, corresponding to a reduction of approximately 61%. In contrast, F1_Zn decreased from 230.5 mg kg^−1^ (12.9%) to 121.3 mg kg^−1^ (7.5%) in JB10, equivalent to a reduction of about 41% (Tables S5 and S6).

These contrasting trends reflected fundamental differences in metal-biochar interactions. Pb readily formed stable complexes with oxygen-containing functional groups and associated strongly with Fe/Mn oxides, resulting in substantial enrichment of Pb in F3 and F4 fractions. Conversely, Zn was governed primarily by pH adjustment, ion exchange, and electrostatic adsorption, with limited formation of stable inner-sphere complexes. Consequently, Zn exhibited weaker redistribution toward highly stable fractions compared with Pb, even under similar amendment intensity.

Overall, the fractionation results clearly demonstrated that Pb and Zn cannot be treated using a uniform immobilization strategy. While Pb was effectively stabilized through organic complexation and pH-driven precipitation processes, Zn remained more sensitive to pH and ion-exchange dynamics, leading to a comparatively higher residual mobility. These findings not only advanced mechanistic understanding of metal-specific behavior in biochar-amended soils but also underscored the necessity of designing remediation strategies tailored to individual target metals in multi-contaminated systems.

#### 3.4.3. Transformation of the exchangeable fraction (F1) and Pb/Zn immobilization index

In evaluating changes in the chemical forms of Pb and Zn following biochar amendment, particular emphasis was placed on the exchangeable fraction (F1), which represents the most mobile and potentially bioavailable pool. Focusing on this fraction enabled a standardized comparison of immobilization efficiency and provided clearer insight into the dominant mechanisms governing the behavior of the two metals.

##### 3.4.3.1 Pronounced reduction of F1_Pb as a function of biochar type and application rate

A substantial decline in Pb associated with the exchangeable fraction was observed across biochar types and application rates (Tables 4 and S5 (SI)). In the control soil (BS), F1_Pb accounted for 464.6 ± 32.5 mg kg^−1^, corresponding to 11.9% of total Pb. Following biochar amendment, F1_Pb progressively decreased to 280.6 ± 20.1 mg kg^−1^ (7.9%) for SB10, 299.4 ± 22.3 mg kg^−1^ (8.4%) for JB10, and reached the lowest level of 182.6 ± 16.5 mg kg^−1^ (5.1%) for TB10.

The magnitude of this reduction exceeded that observed for Zn under comparable conditions, indicating a higher immobilization efficiency for Pb in the biochar-amended system. The effectiveness of F1_Pb reduction increased with application rate, with the 10% (w/w) treatment producing the strongest response, particularly for biochar derived from *Colocasia* stems (TB). This pattern reflected the combined influence of elevated soil pH, abundant surface functional groups, and mineral ash content in promoting Pb complexation and precipitation. Increases in soil pH and the availability of –COOH and –OH groups enhanced Pb^2+^ complexation and favored the formation of Pb carbonates and hydroxides, leading to a marked depletion of the exchangeable fraction.

##### 3.4.3.2 Comparison with Zn–F1: weaker reduction and distinct mechanisms

In contrast to Pb, Zn exhibited a more moderate decrease in the exchangeable fraction, both in relative contribution and absolute concentration. In the control soil, F1_Zn was 230.5 ± 18.6 mg kg^−1^ (12.9%). After biochar amendment, F1_Zn declined to 139.8 ± 13.1 mg kg^−1^ (8.4%) for SB10, 121.3 ± 11.9 mg kg^−1^ (7.5%) for JB10, and 131.6 ± 12.8 mg kg^−1^ (7.8%) for TB10 (Tables 5 and S6 (SI)). Although a reduction was evident at the highest application rate, the extent of F1_Zn depletion remained substantially lower than that of F1_Pb under identical conditions.

This contrast reflected the weaker affinity of Zn^2+^ for stable inner-sphere complexation sites on biochar surfaces compared with Pb^2+^. Zn retention appeared to be primarily associated with pH-dependent adsorption and ion-exchange processes. As a result, Zn was preferentially redistributed toward carbonate-bound or Fe/Mn oxide-associated fractions rather than forming stable organic complexes.

##### 3.4.3.3 Mechanistic comparison of Pb and Zn based on the F1 fraction

By isolating the exchangeable fraction, which best represents environmental mobility and bioavailability, clear differences in Pb and Zn behavior became apparent:

• F1_Pb declined sharply with increasing biochar dose and varied strongly with biochar type, particularly for TB10, demonstrating the susceptibility of Pb to immobilization *via* organic complexation and pH-driven mineral precipitation.

• F1_Zn also decreased but to a lesser extent, indicating that Zn was retained mainly through ion exchange and electrostatic adsorption, with limited formation of stable surface complexes.

These differences reflected intrinsic chemical properties of the two metals, including ionic charge density, hydration energy, and ionic radius, and underscored the necessity of evaluating metal-specific behavior when designing remediation strategies for multi-metal contaminated soils. By concentrating on the exchangeable fraction, this study provided a clearer assessment of the environmentally relevant mobility and bioaccessibility of Pb and Zn, as well as the actual immobilization capacity of different biochars under identical experimental conditions.

### 3.5. Principal component analysis (PCA): linking biochar properties with Pb/Zn fractionation

#### 3.5.1. PCA score and loading patterns

**Fig. 7:**
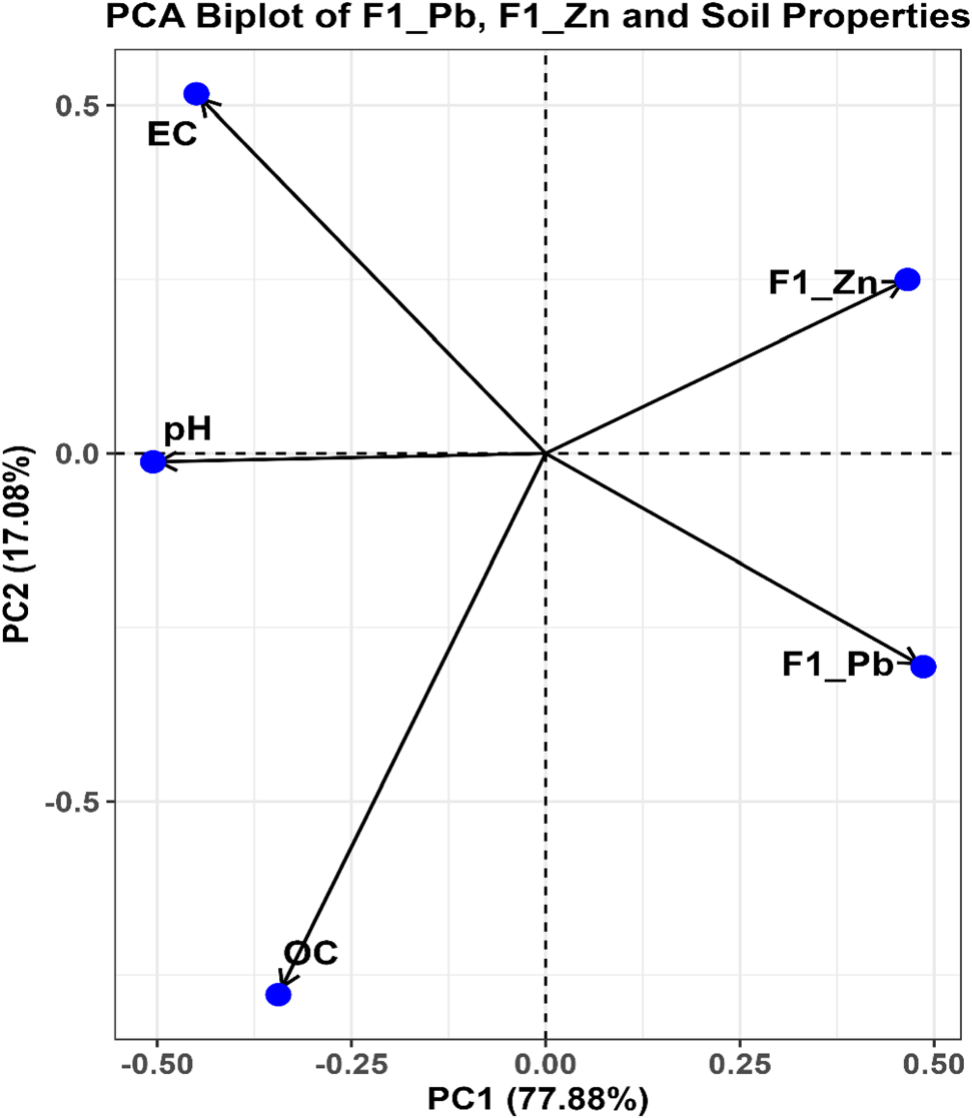
PCA biplot illustrating the relationships between labile Pb and Zn fractions (F1_Pb and F1_Zn) and soil physicochemical properties (pH, EC, and OC). The first two principal components explain 94.96% of the total variance.

Principal component analysis (PCA) was employed to explore multivariate relationships between soil–biochar properties (pH, organic carbon (OC), and electrical conductivity (EC)) and metal mobility, represented by the exchangeable fraction (F1) of Pb and Zn. Prior to analysis, all variables were standardized using *z*-score normalization to ensure equal weighting. The PCA outcomes were summarized in Fig. 7 and Table 7. PCA was applied as an exploratory tool to identify patterns of association among a limited set of variables (pH, OC, EC, F1_Pb, and F1_Zn). It does not incorporate the full range of biochar properties discussed elsewhere in the study.

PCA loadings and explained variance

**Table:** 

(a) PCA loadings of variables on the first two principal components^a^		
Variable	PC1	PC2
F1 (Pb)	**+0.479**	−0.302
F1 (Zn)	**+0.459**	+0.246
pH	**−0.498**	−0.012
EC	**−0.443**	**+0.509**
OC	**−0.339**	**−0.767**

a Bold values indicate strong loadings.

b PCA was performed on *z*-score standardized variables (pH, OC, EC, F1_Pb and F1_Zn). Only the first two principal components are shown as they explain more than 90% of the total variance. PCA results are interpreted as exploratory associations among selected variables and do not provide direct mechanistic evidence.

**Table:** 

(b) Eigenvalues and explained variance^b^			
PC	Eigenvalue	Variance (%)	Cumulative variance (%)
PC1	3.89	77.88	77.88
PC2	0.85	17.08	94.96

The first two principal components (PC1 and PC2) accounted for 94.96% of the total variance, with PC1 explaining 77.88% and PC2 explaining 17.08% (Table 6). The high proportion of explained variance indicates that the selected variables capture a dominant pattern of variation within the dataset; however, this does not imply a complete representation of all controlling factors.

The loading pattern revealed strong positive contributions of F1_Pb (+0.479) and F1_Zn (+0.459) along PC1, whereas pH (−0.498), EC (−0.443), and OC (−0.339) exhibited pronounced negative loadings on the same axis. This opposing orientation suggests an inverse association between soil chemical properties (pH, EC, OC) and the exchangeable fractions of Pb and Zn.

In the score plot, biochar-amended soils were clearly separated from the control treatment. Samples receiving higher application rates, particularly the 10% (w/w) treatments (*e.g.*, TB10 and SB10), clustered toward the negative side of PC1, corresponding to elevated pH, OC, and EC and reduced F1 values. This separation reflects consistent differences among treatments but should be interpreted as a pattern of association rather than a direct mechanistic distinction.

#### 3.5.2. PCA-based interpretation of Pb and Zn immobilization mechanisms

Beyond treatment differentiation, principal component analysis (PCA) provided insight into the relationships between soil chemical conditions and metal mobility. PC1 represented a dominant gradient linking variations in soil properties to changes in the mobility of Pb and Zn. The strong negative loadings of pH, EC, and OC along this axis were associated with lower values of F1_Pb and F1_Zn, indicating reduced metal mobility under conditions related to biochar amendment. This pattern is consistent with the effects of alkalization, increased ionic strength, and organic carbon enrichment in limiting metal availability; however, it should be noted that PCA reflects statistical associations rather than direct causal relationships.

PC2 captured differences in the behavior of Pb and Zn in response to changes in soil conditions. Organic carbon exhibited a strong negative loading (−0.767), whereas EC showed a positive loading (+0.509) (Table 7). Along this axis, F1_Pb displayed a slight negative loading (−0.302), while F1_Zn showed a relatively weak positive contribution (+0.246). These patterns suggest that Pb and Zn may respond differently to variations in soil chemical properties, although the relatively low loading of F1_Zn indicates that this separation should be interpreted with caution. Therefore, PCA results are used to indicate potential differences in controlling factors rather than to define definitive mechanistic pathways.

Overall, PCA provided a quantitative basis for identifying key variables associated with metal mobility. By highlighting pH, OC, and EC as primary factors, PCA served as an intermediate analytical step linking experimental observations to subsequent machine learning analyses, including Random Forest and XGBoost, in which these variables were further evaluated for their predictive importance.

### 3.6. Machine learning models for simulating Pb and Zn fraction transformation

#### 3.6.1. Model performance and reliability assessment

**Fig. 8:**
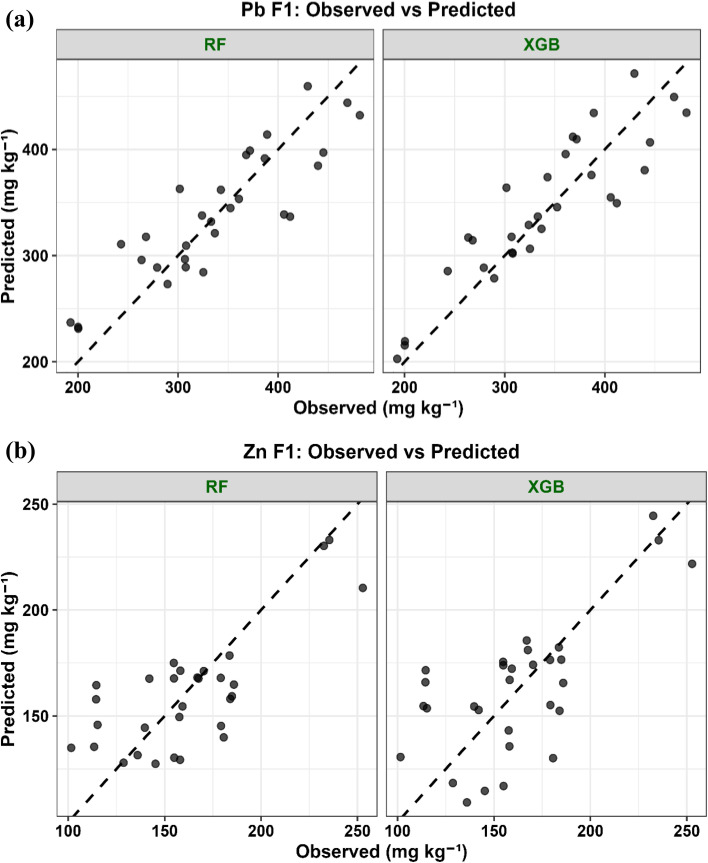
Comparison between observed and predicted concentrations of metals in the exchangeable fraction (F1) for (a) Pb and (b) Zn using the Random Forest (RF) and Extreme Gradient Boosting (XGB) models. The dashed line represents the 1 : 1 line, indicating perfect agreement between observed and predicted values.

**Fig. 9:**
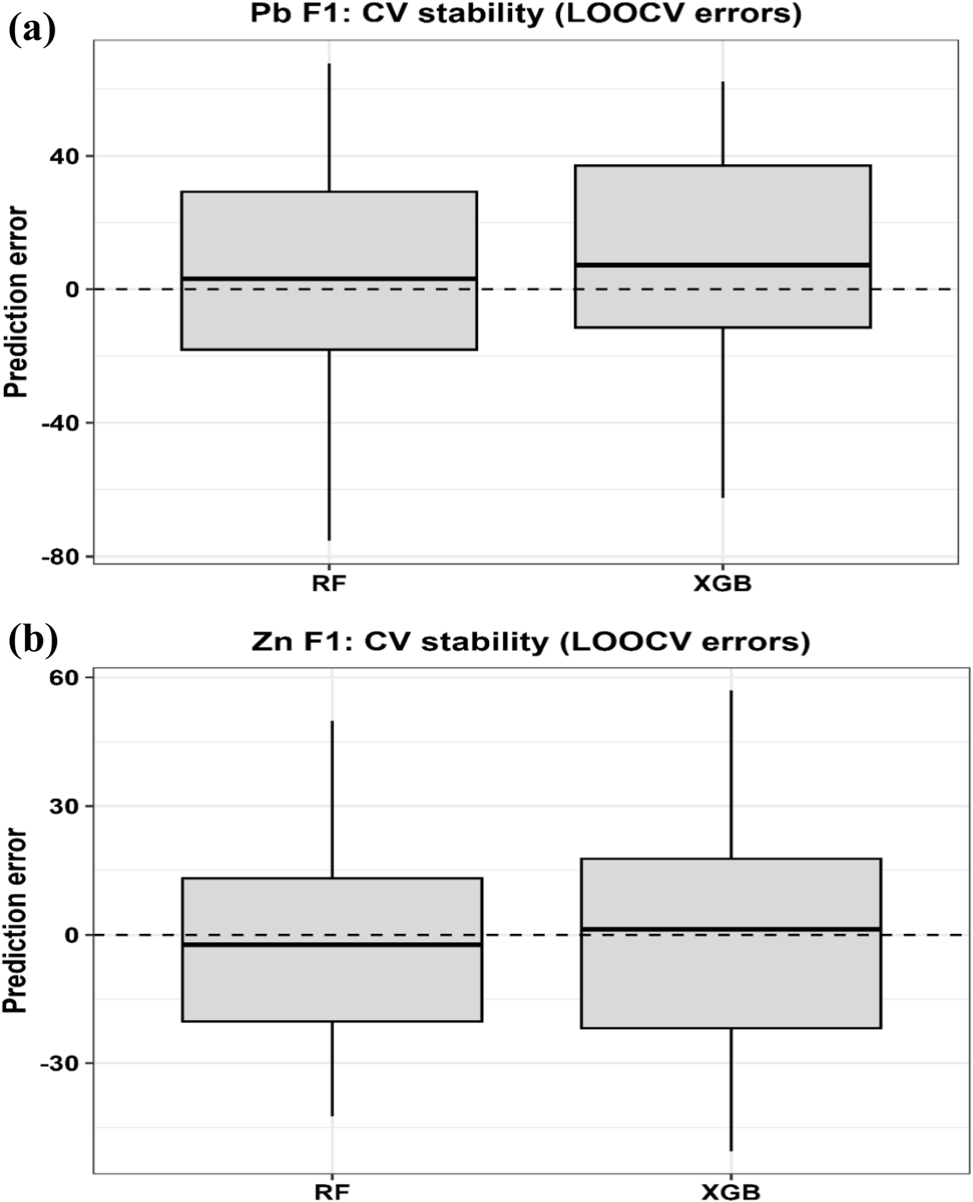
Cross-validation (CV) stability. Distribution of prediction errors obtained from leave-one-out cross-validation (LOOCV) for the exchangeable fraction (F1) of (a) Pb and (b) Zn using the Random Forest (RF) and Extreme Gradient Boosting (XGB) models. The dashed line indicates zero error, corresponding to unbiased predictions.

The application of machine learning in this study is intended to complement, rather than replace, conventional statistical analysis. Ensemble methods such as Random Forest and XGBoost are suitable for capturing nonlinear relationships in complex environmental systems, particularly when interactions among variables are not easily represented by linear models. In this study, two widely used machine learning algorithms, Random Forest (RF) and Extreme Gradient Boosting (XGBoost; XGB), were applied to predict Pb and Zn concentrations across the Tessier sequential extraction fractions (F1–F5). The input data was shown in Tables S7–S9 (in SI). Model performance was evaluated using three statistical indicators, including the coefficient of determination (*R*^2^), root mean square error (RMSE), and mean absolute error (MAE) (Table S10 (SI)). These metrics were complemented by comparisons between observed and predicted values of F1 of Pb and Zn (Fig. 8) and an assessment of model stability based on cross-validation (CV) results of F1_Pb and F1_Zn (Fig. 9).

The application of machine learning in this study is intended to complement, rather than replace, conventional statistical analysis. Ensemble methods such as Random Forest and XGBoost are suitable for capturing nonlinear relationships in complex environmental systems, particularly when interactions among variables are not easily represented by linear models.

Model performance varied across fractions, with XGBoost showing an advantage in fractions governed by stronger nonlinearity, particularly for Pb. In contrast, Random Forest produced more stable predictions for Zn, where controlling factors appeared more distributed. Detailed performance metrics for all fractions are provided in Table S11 (see in SI)

##### 3.6.1.1 Overall comparison between RF and XGBoost

Across all fractions, XGBoost generally produced slightly higher predictive accuracy than RF for fractions governed by pronounced nonlinear behavior, particularly F1_Pb, F4_Pb, and F5_Pb. For example, in the case of F1_Pb, XGBoost achieved an *R*^2^ value of 0.800, exceeding that of RF (*R*^2^ = 0.776), while simultaneously yielding lower prediction errors (RMSE = 34.60 mg kg^−1^ for XGB compared with 36.88 mg kg^−1^ for RF). This performance reflected the strength of XGBoost in capturing nonlinear relationships and complex interactions among soil environmental variables, such as pH, organic carbon (OC), electrical conductivity (EC), and biochar application rate, which exert strong control over the exchangeable metal fraction (F1).

By contrast, RF exhibited comparatively stable and reliable performance for several Zn fractions, notably F1_Zn and F5_Zn, where controlling mechanisms appeared more diffuse and less dominated by a single driving variable. This behavior suggested that RF was well suited to modeling fractionation patterns characterized by broader variance structures and reduced sensitivity to localized nonlinear effects.

Fig. 8 illustrate the relationships between observed and predicted values of the exchangeable fraction (F1) for Pb and Zn obtained from the RF and XGB models. For Pb (Fig. 8), most data points clustered closely around the 1 : 1 line, indicating high predictive accuracy and minimal systematic bias across the entire concentration range. In particular, the XGB model showed improved agreement at higher concentration levels, highlighting its strength in capturing nonlinear relationships governing Pb behavior.

In contrast, predictions for F1_Zn (Fig. 8) exhibited greater dispersion, especially at lower concentration ranges. This pattern reflects the inherently higher mobility of Zn and its stronger sensitivity to variations in soil solution chemistry. Despite this increased variability, both models successfully reproduced the overall trend of the observed data. Collectively, these results demonstrate that RF and, more prominently, XGB provide robust tools for predicting mobile metal fractions in biochar-amended soils. The relationships between observed and predicted values of all fractions (F1–F5) of Pb and Zn are shown in Fig. S1 and S2 in the SI.

Fig. 9 illustrates the stability of the RF and XGB models based on the distribution of prediction errors derived from LOOCV for the exchangeable fraction (F1) of Pb and Zn. For F1_Pb (Fig. 9), prediction errors from both models were symmetrically distributed around zero, indicating the absence of pronounced systematic bias. The RF model exhibited a narrower error spread, suggesting greater stability across individual training iterations, whereas XGB showed a small number of larger deviations at the distribution tails, reflecting higher sensitivity to extreme data points. The stability of the RF and XGB models based on the distribution of prediction errors derived from LOOCV for the five fractions (F1–F5) of Pb and Zn is also shown in Fig. S3 and S4 in the SI.

For F1_Zn (Fig. 9), the error distributions were broader than those observed for Pb, consistent with the higher mobility of Zn and its stronger responsiveness to variations in soil chemical conditions. Nevertheless, the median prediction errors for both RF and XGB remained close to zero, indicating acceptable generalization performance. Overall, these results confirm that LOOCV provides a reliable framework for assessing the robustness and predictive consistency of machine learning models when applied to mobile metal fractions in soil systems.

##### 3.6.1.2 Comparison between Pb and Zn

Model performance differed markedly between the two metals. Overall, predictions for Pb were more accurate than for Zn, particularly for the exchangeable (F1) and residual (F5) fractions. Among all outputs, Pb–F5 exhibited the highest predictive performance, with *R*^2^ values of 0.843 for RF and 0.849 for XGB. This behavior reflects the intrinsic stability of Pb in the residual fraction, where concentrations show limited short-term variability and are less sensitive to fluctuations in soil chemical conditions, thereby facilitating model learning and prediction accuracy.^33^

In contrast, Zn displayed lower *R*^2^ values across most fractions, with particularly weak performance for F2 and F3. For instance, Zn–F2 yielded *R*^2^ values of only 0.074 (RF) and 0.255 (XGB), indicating limited predictive capability. This reduced performance can be attributed to the higher mobility of Zn and its rapid responsiveness to changes in ionic strength and pH, which induce pronounced fluctuations in Zn partitioning among carbonate-bound and Fe/Mn oxide-bound fractions. The stronger immobilization of Pb relative to Zn observed in this study aligns with previous reports,^27,34^ where Pb stabilization was associated with stronger surface interactions, whereas Zn remained more influenced by reversible processes such as adsorption and ion exchange. However, the extent of redistribution observed here appears more pronounced, which may reflect differences in soil properties and biochar mineral composition.

##### 3.6.1.3 Fraction-wise model performance

When examined by individual chemical fractions, distinct performance patterns emerged:

• F1 (exchangeable fraction): this fraction was predicted with relatively high accuracy for both metals, particularly for Pb. The strong performance reflects the clear dependence of F1 on input variables such as pH, EC, and OC, which were consistently identified as influential factors in PCA and feature-importance analyses.

• F2 and F3: both RF and XGB exhibited limited predictive performance (*R*^2^ < 0.3), consistent with the transitional nature of carbonate-bound and Fe/Mn oxide-bound fractions. This trend aligns with the transitional and operationally defined nature of these geochemical pools, which are simultaneously governed by fluctuations in pH, carbonate chemistry, and redox-sensitive mineral phases.^35,36^ Unlike the more stable residual or exchangeable fractions, F2 and F3 are sensitive to background ionic conditions and overlapping non-linear controls, complicating robust quantitative modeling.^37^ Similar challenges in capturing the dynamics of these fractions have been documented in biochar-amended soils; the introduction of biochar induces high temporal and geochemical variability in the F2 and F3 pools by altering the rhizosphere's alkalinity and redox potential.^38,39^ Recent machine-learning investigations further corroborate that carbonate- and oxide-associated metal fractions typically yield lower prediction accuracy compared to other fractions, a direct consequence of their mixed mechanistic controls and extreme sensitivity to multiple interacting soil variables.^36,40^

• F4 and F5: model performance improved substantially, especially for Pb. The higher stability and lower variability of organic-bound and residual fractions reduced noise in the data, thereby enhancing model predictability.

Despite these patterns, model performance was not uniform across all fractions. Higher consistency and predictive reliability were observed for Pb–F1 and Pb–F5, whereas several fractions, particularly Pb–F2, Pb–F3, Zn–F2, and Zn–F3, exhibited substantially lower predictive stability. This variability reflects the complex and transitional nature of these fractions, which are governed by multiple interacting and non-linear soil processes that are difficult to capture robustly within the current modeling framework.

Consequently, these differences limit the applicability of the models as fully quantitative predictors across all fractions. Rather than constituting a validated predictive framework, the machine-learning results should be interpreted as exploratory, providing supportive insights into variable importance and system behavior. In this context, the primary value of the ML analysis lies in identifying dominant controlling factors and complementing experimental observations, rather than delivering precise predictive outputs across all geochemical fractions.

##### 3.6.1.4 Assessment of overfitting

**Fig. 10:**
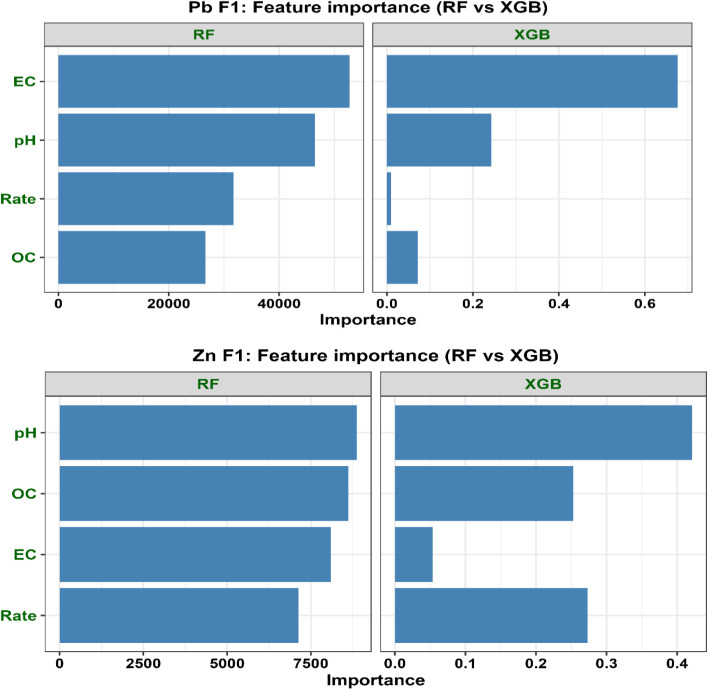
Comparison of feature importance derived from RF and XGB models for Pb and Zn (F1 fraction), showing the relative influence of soil pH, OC, EC, and biochar application rate on metal mobility.

A key concern raised by reviewers relates to the potential risk of overfitting in ML-based analyses. In the present study, no clear evidence of overfitting was observed, supported by three lines of evidence:

(i) Consistency between training and cross-validation results, as illustrated by the CV stability analysis (Fig. 10), where *R*^2^ and RMSE values varied only marginally across folds.

(ii) Observed *versus* predicted plots (Fig. 9) showed data points distributed symmetrically around the 1 : 1 line, without systematic deviations across concentration ranges.

(iii) Model performance remained moderate for chemically complex fractions (F2 and F3), indicating that the models captured realistic system complexity rather than memorizing the dataset.

Taken together, these findings align with recent studies employing RF and XGBoost for PTEs prediction in soils, which emphasize cross-validation as a critical step for ensuring model generalization and robustness.^36^

#### 3.6.2. Feature importance analysis using Random Forest and XGBoost

Feature importance analysis was conducted using Random Forest (RF) and Extreme Gradient Boosting (XGBoost) to identify the dominant factors governing the redistribution of Pb and Zn among chemical fractions in biochar-amended soils (Fig. S5 and S6 in SI). Beyond simple variable ranking, this analysis aimed to extract mechanistic signals from the ML models, thereby elucidating metal-specific controls and differences between labile and stable fractions. This approach highlights the novelty of ML as an interpretive tool rather than a purely predictive framework in PTEs immobilization studies.

##### 3.6.2.1 Relative importance of input variables

Results from both RF and XGBoost consistently identified soil pH, organic carbon content (OC), electrical conductivity (EC), and biochar application rate as influential predictors of metal concentrations across Tessier fractions (see Fig. S5 and S6 in SI). Nevertheless, the relative contribution of each variable varied substantially depending on the target metal and the fraction considered.

Across all models, pH ranked among the most influential variables for both Pb and Zn, underscoring the central role of acid–base conditions in controlling metal solubility, ionic speciation, and precipitation behavior in soil systems. Organic carbon (OC) contributed strongly to model performance, while surface-related interpretations are supported by physicochemical characterization (*e.g.*, BET surface area), which was not included as a predictor in the ML models. EC captured the effect of soluble ions and background electrolyte conditions, which influence ion exchange and electrostatic interactions. The biochar application rate acted as a scaling factor that modulated the intensity of soil-biochar interactions, particularly under higher amendment levels.

##### 3.6.2.2 Mechanistic differentiation between Pb and Zn inferred from ML

A key outcome of the feature importance analysis was the clear contrast between Pb and Zn. For Pb, variables associated with pH and EC were consistently ranked higher than OC and rate, especially for the exchangeable fraction (F1) (Fig. 10). This pattern suggests that Pb immobilization was predominantly governed by pH-driven mechanisms, including hydroxide and carbonate precipitation, as well as ion exchange with alkaline and alkaline-earth cations released from biochar ash.

In contrast, Zn showed a relatively stronger dependence on OC, particularly in fractions beyond F1 (Fig. 10), suggesting a greater contribution of surface adsorption and weaker complexation processes. These ML-derived patterns are consistent with established geochemical behavior, where Pb tends to form stronger inner-sphere complexes and low-solubility precipitates, whereas Zn remains more sensitive to electrostatic interactions and ion exchange processes.

For other fractions of Pb and Zn (such as F2–F5), the feature importance results are shown in Fig. S5 and S6 in the SI.

Overall, the feature importance results demonstrate that RF and XGBoost not only reproduced experimental trends but also provided quantitative support for metal-specific immobilization pathways. This strengthens the mechanistic interpretation of Pb and Zn stabilization and illustrates how explainable ML can bridge complex experimental datasets with process-level understanding.

In contrast, for Zn, organic carbon (OC) exhibited relatively higher importance alongside pH. This pattern reflects the inherently higher mobility of Zn^2+^ and the prominent role of oxygen-containing surface functional groups on biochar in governing Zn retention through adsorption and weak surface complexation. Such behavior is consistent with recent findings indicating that Pb tends to form more stable mineral phases, whereas Zn is predominantly retained *via* surface adsorption and less stable binding mechanisms.

##### 3.6.2.3 Labile *versus* stable fractions

Feature importance analysis further revealed a clear distinction between labile and stable chemical fractions. For the more mobile fractions (F1), variables such as pH and EC consistently ranked higher, highlighting the dominant influence of the immediate soil chemical environment on metal bioavailability and short-term mobility. In contrast, for the more stable fractions (F3–F5), and OC gained relative importance, indicating that long-term stabilization was increasingly governed by surface adsorption, complexation, and incorporation of metals into biochar-associated solid phases.

The systematic shift in variable importance from pH/EC to OC when moving from labile to stable fractions provides quantitative evidence that metal immobilization evolved from rapid solubility control toward longer-term stabilization mechanisms, rather than representing a purely transient decrease in metal availability.

##### 3.6.2.4 Consistency and complementarity between RF and XGBoost

Both RF and XGBoost exhibited broadly consistent patterns in variable ranking, particularly for dominant predictors such as pH and OC, thereby reinforcing the robustness of the mechanistic interpretations. Nevertheless, subtle differences were observed between the two algorithms. XGBoost tended to emphasize a smaller number of dominant variables with higher contrast, whereas RF distributed importance more evenly across multiple predictors.

The complementary behavior of RF and XGBoost reduced the likelihood of model-specific bias and confirmed that the observed feature importance patterns were not artifacts of a single algorithm, but rather reflected underlying geochemical controls within the soil-biochar-metal system.

Fig. 10 illustrates the relative contributions of environmental variables and treatment parameters to the prediction of the exchangeable fraction (F1) of Pb and Zn. For F1_Pb (Fig. 10), EC and pH emerged as the most influential variables in both models, indicating that exchangeable Pb was strongly regulated by ionic strength and acid-base conditions. Biochar application rate and OC played secondary roles, contributing to Pb immobilization through adsorption and surface complexation processes.

For F1_Zn (Fig. 10), pH and OC dominated the importance rankings, reflecting the higher mobility of Zn and its strong dependence on adsorption onto organic matter and biochar functional surfaces. Differences in the relative rankings between RF and XGBoost suggest that each algorithm captured variable interactions in distinct ways; however, both converged on the central role of pH in controlling the behavior of exchangeable metal fractions.

#### 3.6.3. Model interpretation using SHAP and PDP: controlling mechanisms of the labile (F1) fractions of Pb and Zn

**Fig. 11:**
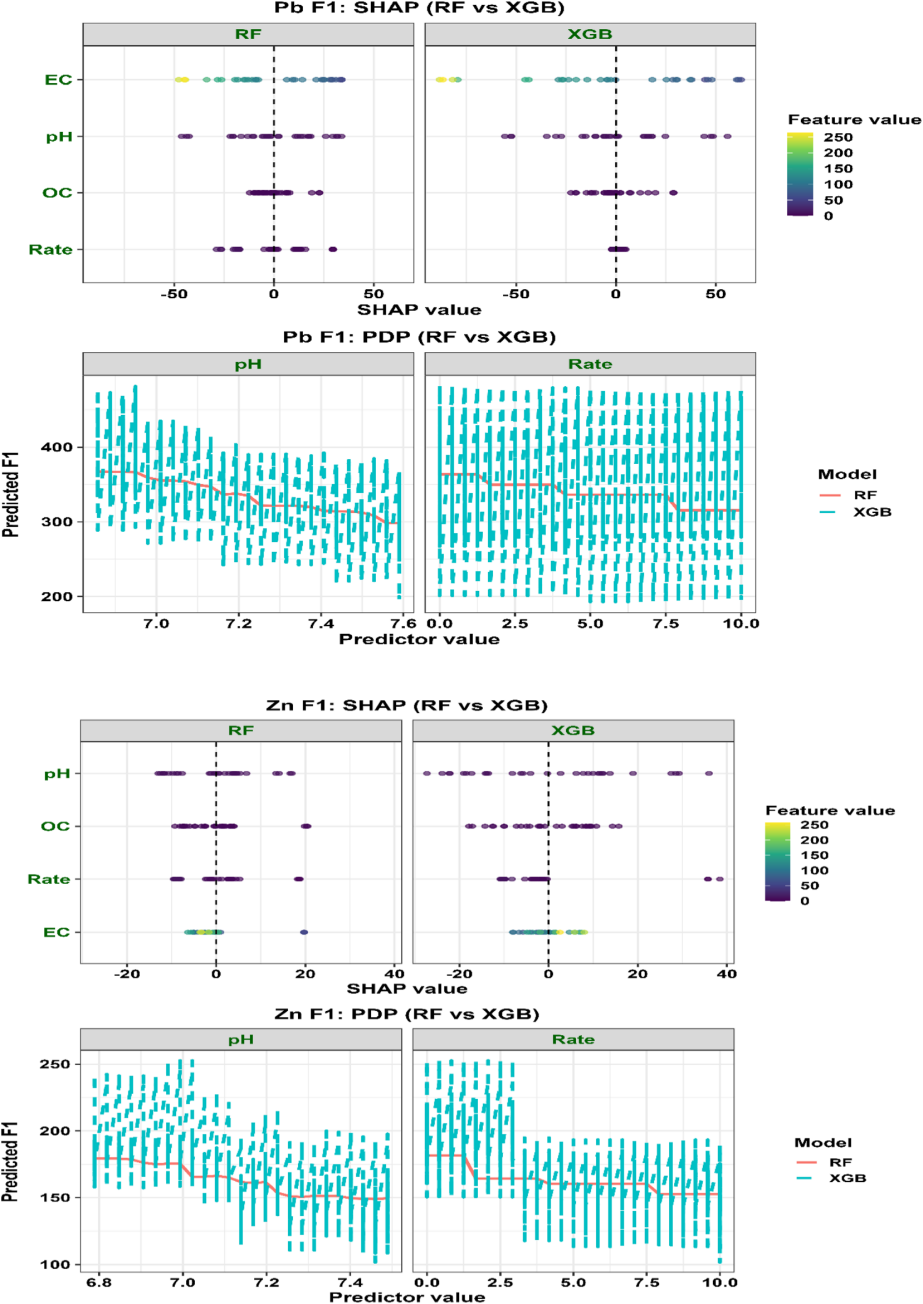
Interpretability analysis of the exchangeable fraction (F1_Pb) of Pb and Zn (F1_Zn) using SHapley Additive exPlanations (SHAP) and partial dependence plots (PDP) based on random forest (RF) and extreme gradient boosting (XGB) models. SHAP plots illustrate both the magnitude and direction of the effects of input variables (pH, organic carbon (OC), electrical conductivity (EC), and biochar application rate) on model predictions, whereas PDPs describe the nonlinear relationships between the most influential variables and the predicted F1 metal concentrations.

To enhance model transparency and mechanistic interpretability, SHapley Additive exPlanations (SHAP) combined with partial dependence plots (PDPs) were applied to elucidate both the relative importance and directional effects of key input variables (pH, EC, OC, and biochar application rate) on predictions of the labile fraction (F1) of Pb and Zn (Fig. 11). Unlike global performance metrics (*R*^2^, RMSE, MAE), SHAP enabled direct quantification of how individual variables contributed to model outputs, thereby linking machine-learning predictions with underlying soil–chemical processes governing metal behavior.

##### 3.6.3.1 F1_Pb: dominant control by pH and EC, consistently captured by RF and XGBoost

SHAP results for F1_Pb (Fig. 11) indicated that pH and EC exhibited the largest absolute SHAP values in both RF and XGBoost models, clearly exceeding the contributions of OC and biochar application rate. Data points clustered predominantly on the negative SHAP side as pH increased, demonstrating that higher pH exerted a strong suppressive effect on the exchangeable Pb fraction, which represents the most mobile and environmentally sensitive form of Pb.

This pattern closely mirrored the experimental observations, where F1_Pb declined markedly from 464.6 mg kg^−1^ (11.9%) in the control soil (BS) to 182.6 mg kg^−1^ (5.1%) in the TB10 treatment, concurrent with an increase in soil pH from 6.84 to 7.55 following biochar amendment. Such consistency supports a mechanistic interpretation in which Pb immobilization was governed primarily by (i) reduced Pb^2+^ solubility under elevated pH conditions, (ii) enhanced precipitation of Pb as carbonate and hydroxide phases, and (iii) stronger specific adsorption onto Fe/Mn oxides and oxygen-containing functional groups on biochar surfaces.

Electrical conductivity also contributed substantial negative SHAP values, particularly in mineral-rich treatments such as TB, highlighting the role of released alkaline and alkaline-earth cations (*e.g.*, Ca^2+^, Mg^2+^, K^+^) in promoting ion-exchange processes and stabilizing Pb in less mobile forms. PDP analysis for F1_Pb (Fig. 11) further reinforced this interpretation by revealing a pronounced inverse relationship between soil pH and F1_Pb, with a sharp decrease occurring beyond a threshold of approximately pH 7.1 and a tendency toward stabilization at pH values above ∼7.4. The threshold is model-derived within the studied pH domain, not universal.

Together, the SHAP and PDP results consistently indicated that pH-related processes, modulated by ionic strength and mineral-derived cations, were key factors associated with the exchangeable Pb fraction in biochar-amended soils, in line with recent mechanistic studies.

##### 3.6.3.2 F1_Zn: dispersed, condition-dependent, and less stable responses compared with Pb

In contrast to Pb, SHAP analysis for F1_Zn (Fig. 11) displayed smaller absolute SHAP values and a more scattered distribution across input variables. This pattern reflects the inherently higher mobility of Zn^2+^ and its weaker tendency to form stable associations within the soil–biochar system. Although pH retained a negative SHAP trend (higher pH corresponding to lower F1_Zn), its influence remained noticeably weaker than that observed for Pb.

This behavior aligned with experimental observations, where F1_Zn decreased from 230.5 mg kg^−1^ (12.9%) in the control soil to 131.6 mg kg^−1^ (7.8%) in TB10, indicating a more limited immobilization of Zn in the exchangeable fraction under identical treatment conditions. OC and application rate exhibited SHAP values fluctuating around zero in both models, suggesting a minor contribution of organic complexation to F1_Zn control. PDPs for F1_Zn (Fig. 11) revealed a gradual decline in F1_Zn with increasing pH and biochar dose, albeit with shallow slopes and greater dispersion, pointing to immobilization mechanisms dominated by conditional processes such as pH regulation and ion exchange rather than strong inner-sphere complexation. Such patterns are consistent with recent studies describing Zn as a highly mobile metal whose response to biochar amendments depends strongly on soil physicochemical conditions rather than specific chemical binding pathways.^36^

##### 3.6.3.3 Methodological implications: SHAP and PDP as a bridge between ML and geochemical mechanisms

The consistent identification of pH and EC as dominant drivers for F1_Pb, contrasted with weaker control for F1_Zn, suggests distinct controls on metal redistribution. These patterns indicate that the models captured meaningful associations with soil geochemical conditions, without directly resolving the underlying mechanisms. In this context, SHAP and PDP served as an effective bridge linking experimental evidence, multivariate analysis (PCA), and machine learning outputs. These tools also provided a rational basis for feature selection in subsequent ML modeling.

Model interpretation relied on feature importance and SHAP analysis, allowing identification of consistent controlling variables across fractions. These findings were compared with PCA results to ensure consistency between data-driven and statistical approaches.

#### 3.6.4. ML-assisted interpretation of Pb and Zn immobilization patterns

Integrating interpretable machine-learning models (random forest and XGBoost coupled with SHAP and PDP) with experimental observations provided a coherent framework for clarifying the processes governing Pb and Zn immobilization in biochar-amended soils. Rather than operating as opaque predictive tools, the ML models exhibited consistent agreement with fractionation data and material characterization, supporting the interpretation of patterns inferred from classical geochemical analyses.

##### 3.6.4.1 pH-driven immobilization as the dominant control in the Pb–Zn-biochar system

Across all ML outputs, soil pH emerged as the primary variable controlling the exchangeable fraction (F1) of both Pb and Zn. In RF and XGBoost models alike, pH carried the largest absolute SHAP values for F1_Pb and F1_Zn, with a clear negative contribution, indicating systematic suppression of metal mobility as pH increased. This pattern was echoed by PCA, where pH loaded opposite to F1 along the main variance axis.

Experimental trends followed the same direction. Raising soil pH from 6.84 in the control (BS) to 7.55 in TB10 coincided with a pronounced decline in F1_Pb from 464.6 mg kg^−1^ (11.9%) to 182.6 mg kg^−1^ (5.1%), whereas F1_Zn decreased more moderately from 230.5 mg kg^−1^ (12.9%) to 131.6 mg kg^−1^ (7.8%). These parallel responses point to shifts in the soil chemical environment, particularly proton activity, as the main force driving metals away from the exchangeable pool through reduced solubility, enhanced surface retention, and secondary precipitation. While pH-controlled immobilization has been widely reported, the present ML framework quantified its dominance relative to other variables, moving beyond qualitative description.^41^

##### 3.6.4.2 Divergent immobilization pathways for Pb and Zn

A second pattern identified by the ML analysis concerned the contrasting behavior of Pb and Zn under identical amendment conditions. SHAP and PDP analyses indicated a stronger and more coherent response of Pb to changes in pH and EC, whereas Zn responses were weaker and more dispersed.

For Pb, ML highlighted additional contributions from EC and OC alongside pH, consistent with specific adsorption and stable complexation between Pb^2+^ and oxygen-containing functional groups (–COO^−^, phenolic OH) on biochar surfaces, as well as interactions with Fe/Mn oxides. This interpretation was supported by fractionation data, where Pb accumulated in F3 and F4, with F4_Pb increasing from 113.4 mg kg^−1^ (2.9%) in BS to 211.6 mg kg^−1^ (5.9%) in TB10, indicating chemically specific and persistent immobilization.

Zn displayed a different pattern. Contributions from OC and EC to F1_Zn were comparatively minor, while pH exerted a more gradual, condition-dependent influence. Such behavior suggests retention dominated by non-specific processes, including electrostatic adsorption and ion exchange, rather than stable organic complexation. This interpretation matched experimental observations, where F4_Zn remained near 4–5% and showed little response to increasing biochar dose, underscoring the limited role of strong organic binding for Zn.^36^ These distinctions are consistent with recent classifications of Pb as having a high affinity for organic matter and oxide surfaces, while Zn remains more mobile and pH-sensitive.^42^

##### 3.6.4.3 Machine learning as a mechanistic lens rather than a black box

From a methodological standpoint, the combined use of SHAP, PDP, and PCA demonstrated that ML can serve as an interpretable, data-driven framework rather than a black-box predictor. Model outputs reinforced conclusions drawn from Tessier fractionation, while adding quantitative resolution regarding the relative influence of controlling factors.

ML identified statistical associations between soil properties (pH, EC, OC) and the redistribution behavior of Pb and Zn, supporting feature selection and informing future optimization of remediation strategies.^36^ This approach reflects a broader shift in environmental research toward mechanism-informed machine learning, where predictive performance and process understanding advance together.^42^

It should be noted that the experimental design primarily evaluates the combined effects of biochar type and application rate, and does not fully isolate the influence of interacting variables such as pH, organic carbon, and mineral composition. Although multivariate analysis (PCA) and machine learning approaches (RF and XGBoost) were applied to identify dominant controlling factors, these methods infer associations rather than establish causality. Therefore, the observed relationships should be interpreted as indicative trends. Future studies employing fully factorial experimental designs and multi-site sampling are recommended to better resolve interaction effects and enhance the general applicability of the findings.

### 3.7. Proposed mechanisms of Pb and Zn immobilization in soil after biochar incubation

The immobilization of Pb and Zn in amended soils arises from a set of interacting processes governed by changes in soil chemistry following biochar incorporation. In this work, interpretation is grounded in fractionation results (F1–F5), together with measured variations in pH, EC, and OC, and supported by FT-IR and SEM-EDS characterization. The discussion below remains evidence-based and avoids attributing specific molecular-scale mechanisms beyond what the available data can support.

#### 3.7.1 Adsorption-related processes

Adsorption likely contributed to the redistribution of metals away from labile pools. A consistent decline in the exchangeable fraction (F1), for example from 11.9% in the control to 5.1% in TB10, suggests a substantial reduction in readily mobile species. This shift is interpreted as increased association of Pb and Zn with solid surfaces rather than direct confirmation of adsorption mechanisms at the molecular level.

The porous structure observed in SEM images, particularly for SB and TB, provides a structural basis for such interactions. In parallel, FT-IR spectra identified oxygen-containing functional groups (*e.g.*, –OH and –COOH), which have been widely reported to participate in metal binding,^32,43^ although their specific role in this system cannot be directly resolved. Electrostatic attraction and weak physicochemical interactions, such as van der Waals forces, may further contribute to metal retention on biochar surfaces.^14,44^ The contribution of these interactions is inferred from the coexistence of surface functionality and fraction changes rather than from direct observation of metal–surface bonding.

#### 3.7.2 Surface complexation

Surface complexation is generally considered a stronger mode of interaction compared with purely electrostatic adsorption, although its relative contribution cannot be directly quantified in the present system. In this study, its involvement is inferred from FT-IR features and the increase in the organic-bound fraction (F4), particularly for Pb.

Functional groups such as carboxyl, carbonyl, and hydroxyl moieties have been reported to coordinate metal ions and form relatively stable surface complexes.^45,46^ Rather than assigning a specific coordination structure, the increase in F4 is interpreted as reflecting stronger surface-associated interactions involving these functional groups.

Differences between Pb and Zn behavior become evident at this stage. Pb exhibited a more pronounced shift toward less labile fractions, whereas Zn remained more distributed in relatively mobile forms. This contrast reflects fundamental differences in aqueous chemistry and coordination behavior. Pb^2+^, with a lower first hydrolysis constant, more readily forms hydrolyzed species under mildly alkaline conditions and interacts with deprotonated functional groups. Within the HSAB framework, Pb^2+^ behaves as a borderline soft acid with stronger affinity for oxygen-donor ligands. In contrast, Zn^2+^ retains stronger hydration and more frequently participates in outer-sphere associations, which partly explains its weaker transition toward stable fractions.^47^ These interpretations remain qualitative, as bonding configurations were not directly resolved.

#### 3.7.3 Ion exchange

Ion exchange may also contribute to metal stabilization. Changes in soil EC following biochar addition indicate increased ionic strength, which can influence competitive interactions at exchange sites. The observed reduction in F1 fractions is consistent with the possibility of partial replacement of Pb^2+^ and Zn^2+^ by cations such as Ca^2+^ and Mg^2+^ released from biochar,^48,49^ although direct quantification of exchange reactions was not performed.

The efficiency of ion exchange depends on surface charge characteristics, pore structure, and solution composition, all of which were altered following amendment.^50^ Therefore, this mechanism is interpreted as a contributing process rather than a dominant or independently verified pathway.

#### 3.7.4 Precipitation-related processes

Biochar application increased soil pH, shifting conditions toward neutral to mildly alkaline ranges and influencing both metal speciation and solubility. An increase in carbonate-associated Pb (F2) was observed with increasing amendment rate. Higher pH conditions reduced metal solubility and may promote processes associated with carbonate-bound fractions, as reflected by the increase in F2. However, this should not be interpreted as direct evidence of specific carbonate precipitation (*e.g.*, PbCO_3_), as the Tessier extraction defines operational fractions rather than discrete mineral phases.

SEM-EDS analysis identified Ca, Mg, and P in the biochar matrix, particularly in TB.^51,52^ FT-IR spectra further indicated phosphate-related functional groups. These observations support the possibility of mineral-associated stabilization pathways, including carbonate or phosphate interactions. However, discrete mineral phases were not directly confirmed (*e.g.*, by XRD or XPS).

At higher pH, deprotonation of functional groups enhances negative surface charge, strengthening cation retention and potentially facilitating the formation of sparingly soluble species.^20^ The formation of metal hydroxides under alkaline conditions may also contribute to stabilization.^51,53^ The presence of such pathways is inferred from geochemical conditions and elemental composition rather than direct mineral identification.

Soil pH operates as a central control across these processes, regulating both surface reactivity and metal speciation.

#### 3.7.5 Integrated interpretation of Pb and Zn behavior

The redistribution patterns of Pb and Zn can be interpreted within a multi-process framework in which adsorption, surface complexation, ion exchange, and precipitation-related processes operate concurrently rather than independently. Pb showed a clear transition toward less labile fractions (F3–F4), indicating stronger association with solid phases and possible involvement of precipitation-related processes. In contrast, Zn exhibited a more limited shift, suggesting that reversible interactions such as adsorption and ion exchange remained more influential.

These observations are consistent with previous studies reporting stronger stabilization of Pb relative to Zn in biochar-amended systems, although the extent of redistribution depends on soil properties and biochar composition. Overall, immobilization behavior is metal-specific and governed by the interplay between intrinsic chemical properties and the modified soil environment.

#### 3.7.6 Mechanistic limitations

While the proposed mechanisms are supported by fractionation results and physicochemical characterization (FT-IR, SEM-EDS, BET), these approaches provide indirect evidence of immobilization pathways. Sequential extraction is operationally defined, and therefore the assignment of metals to specific fractions should be interpreted with caution when linking to discrete mechanisms. Direct identification of metal binding forms would require advanced spectroscopic techniques (*e.g.*, XPS, or synchrotron-based analyses).

**Fig. 12:**
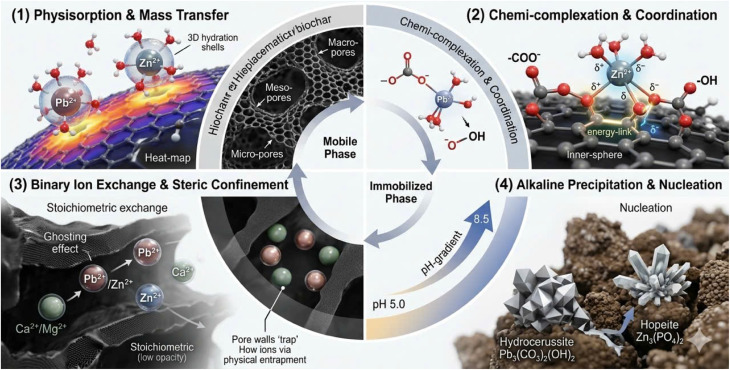
Schematic illustration of the proposed mechanisms for Pb^2+^ and Zn^2+^ immobilization in biochar-amended soil.

A schematic diagram summarizing these interconnected immobilization pathways is provided in Fig. 12, which shows how ion exchange, surface complexation, and precipitation reactions work in concert to contribute to the redistribution of Pb and Zn from mobile fractions (F1–F2) to more stable fractions (F3–F5), thus reducing their mobility and bioavailability in the soil system.

While the mechanistic interpretations proposed in this study are supported by physicochemical characterization (*e.g.*, FT-IR, SEM-EDS, and BET) and interpretable ML analysis, it is acknowledged that these approaches provide indirect evidence of metal immobilization mechanisms. Advanced spectroscopic techniques such as X-ray diffraction (XRD), X-ray photoelectron spectroscopy (XPS), or synchrotron-based analyses could provide more direct confirmation of metal binding forms and mineral phases. The integration of such techniques represents an important direction for future research.

### 3.8. Environmental implications and practical relevance

#### 3.8.1. Ecological risk mitigation through control of labile metal fractions

Assessment of remediation efficiency for metal-contaminated soils benefits from an emphasis on changes in labile metal pools, particularly the exchangeable fraction (F1), rather than reliance on total metal concentrations alone. Within the Tessier sequential extraction framework, F1 represents the most mobile and readily bioavailable metal pool and is closely linked to acute toxicity, plant uptake, and ecological risk.^54,55^ Previous studies have consistently associated labile fractions with metal bioavailability and environmental risk in soils.^56^

In the present system, biochar amendment markedly reduced F1_Pb and F1_Zn while promoting redistribution toward more stable fractions. This shift in chemical speciation points to a reduction in ecological risk driven by changes in metal binding forms rather than simple dilution effects. Comparable decreases in F1_Pb and F1_Zn following biochar application have been reported in contaminated soils, together with reduced metal mobility.^33^ A stronger response was observed for Pb than for Zn, consistent with the geochemical behavior of Pb, which favors specific adsorption, stable complexation, and precipitation processes to a greater extent than Zn.^57,58^

#### 3.8.2. Practical implications for post-mining soils and contaminated agricultural land

These findings carry direct relevance for post-mining soils, tailings-affected areas, and agricultural soils subject to long-term Pb/Zn contamination. Such systems commonly exhibit low pH and a predominance of metals in mobile chemical forms. A growing body of evidence has demonstrated that biochar amendments not only decrease metal bioavailability but also improve soil fertility, water retention, and plant performance, thereby supporting soil rehabilitation and agricultural reuse in mining-impacted regions.^16,59,60^

#### 3.8.3. Implications for biochar selection and application rate

The observed responses underline that biochar does not function as a universal remedy and should be selected according to the target metal and site-specific soil conditions. Biochars with elevated pH and electrical conductivity tend to favor Pb immobilization through acid neutralization and precipitation-driven pathways, whereas Zn retention relies more strongly on surface adsorption and complexation with oxygen-containing functional groups.^46,61^ Synthesis-oriented reviews have likewise emphasized the importance of condition-specific material design to optimize metal stabilization outcomes.^20^ Under laboratory conditions, the 10% (w/w) application rate produced the most pronounced reductions in F1_Pb and F1_Zn field implementation. However, it requires additional consideration of economic feasibility, biomass availability, and potential impacts on soil physical properties.

### 3.9. Limitations and future perspectives

The 30 days incubation captures early-stage shifts in metal fractions but leaves out slower processes such as biochar aging, surface reorganization, and gradual redistribution in soil matrices. These longer-term changes may alter both the strength and persistence of Pb and Zn stabilization, especially as functional groups evolve and mineral associations develop over time.

Field complexity is also absent. Wet–dry cycles, microbial turnover, root activity, and nutrient fluxes continuously reshape soil chemistry and can redirect biochar–metal interactions in ways that laboratory systems cannot fully reproduce. Extrapolation to real soils should therefore remain cautious.

From a modeling standpoint, the dataset size (*N* = 10) constrains the scope of machine learning. Ensemble models (random forest and XGBoost) were chosen for their tolerance to small datasets and nonlinear structure, yet the risk of overfitting cannot be removed entirely. Cross-validation and multi-metric evaluation (*R*^2^, RMSE, MAE) were used to stabilize model assessment and avoid dependence on a single metric.

Interpretation was treated conservatively. Feature importance and SHAP outputs were only retained when consistent across algorithms and aligned with independent statistical structure, particularly PCA. This cross-checking step helps filter out model-specific artifacts and anchors the analysis in reproducible patterns rather than isolated signals. Even so, sensitivity to data structure and limited transferability remain inherent constraints, and the identified relationships should be viewed as indicative rather than definitive.

While the mechanistic interpretations proposed in this study are supported by physicochemical characterization techniques (*e.g.*, FT-IR, SEM-EDS, and BET) in combination with interpretable machine learning analysis, it is important to recognize that these approaches provide indirect evidence of metal immobilization mechanisms. Direct identification of metal binding forms and mineral phases at the molecular or crystallographic level remains unresolved. Advanced spectroscopic techniques, including X-ray diffraction (XRD), X-ray photoelectron spectroscopy (XPS), and synchrotron-based analyses, would enable more definitive characterization of these processes. The integration of such techniques represents a critical direction for future research and would substantially enhance mechanistic resolution.

Future work should focus on longer-term incubation experiments and field-scale validation to better assess the durability of immobilization under realistic environmental conditions. The interactions among aged biochar, soil minerals, and natie organic matter warrant further investigation, particularly in biologically active systems.

In addition, future research should extend the current framework to more complex environmental scenarios, including multi-contaminant systems and a broader diversity of soil types, in order to evaluate the generality and transferability of the observed metal fractionation behavior across heterogeneous conditions. Furthermore, the integration of larger datasets with hybrid mechanistic–machine learning approaches is expected to improve predictive reliability and support the development of more universally applicable remediation strategies.

## 4 Conclusion

Biochars derived from three feedstocks modified the chemical environment of a Pb–Zn contaminated soil during a 30 days incubation. Changes in pH, organic carbon, and ionic composition led to a redistribution of metals among Tessier fractions rather than a reduction in total concentrations, with exchangeable Pb and Zn decreasing and less mobile fractions increasing. Metal stabilization pathways differed according to biochar type and application rate. Ash-rich biochars enhanced Pb immobilization primarily through alkalinity-driven mechanisms, whereas carbon-rich materials were more strongly associated with Zn redistribution, likely through surface-mediated interactions. These findings indicate that metal stabilization is governed by distinct, metal-specific mechanisms rather than a uniform immobilization process. Machine learning analysis provided a quantitative framework for interpreting metal fractionation trends. Soil pH, electrical conductivity, organic carbon, and amendment rate emerged as key predictors of metal partitioning, with their relative importance differing between Pb and Zn. The modeling results complemented the experimental observations and strengthened the interpretation of governing factors.

The findings remain constrained to a single soil system, one production condition, and short-term incubation, and extrapolation beyond these conditions should be approached with caution. Long-term field validation is required to assess the stability of metal immobilization under natural conditions, including aging effects, environmental variability and diverse biochar production parameters to establish more universal stabilization frameworks.

Overall, the results suggest that biochar selection strategies can benefit from considering both metal-specific behavior and soil chemical context, establishing a basis for more targeted and mechanism-informed approaches to soil remediation.

## Author contributions

Truong Xuan Vuong conceived and designed the study, performed the experiments, analysed and interpreted the data, and wrote the original draft of the manuscript. Ha Ngan Nguyen, Thi Thao Truong, Thi Thu Ha Pham, Thi Thu Thuy Nguyen, Thi Tam Khieu, and Thanh Phuong Phan, contributed to experimental work, data collection, and data analysis. The Chinh Pham, Xuan Thang Dam contributed to the review and editing of the manuscript. All authors discussed the results, reviewed the manuscript, and approved the final version for publication.

## Conflicts of interest

The authors declare no conflicts of interest.

## Supplementary Material

RA-016-D6RA01444E-s001

## Data Availability

All data obtained have been included in the manuscript and the supplementary information (SI) and are available from the corresponding author upon reasonable request. Supplementary information: ICP-MS operating conditions and analytical validation (Table S1), and two-way ANOVA results for soil properties and metal fractions (Tables S2, S4 and S6). Detailed distributions of Pb and Zn among Tessier fractions (F1–F5) under different biochar treatments are provided (Tables S3 and S5). Machine learning datasets and input variables are presented in Tables S7–S9, while model performance and feature importance analyses are summarised in Tables S10–S12. Additional figures illustrate model validation, prediction accuracy, cross-validation stability, and feature importance for Pb and Zn fractions (Fig. S1–S6). See DOI: https://doi.org/10.1039/d6ra01444e.
