# Supplementary material for: Comparative immobilization of labile Pb and Zn fractions in contaminated soil using jackfruit seed-, sugarcane bagasse-, and taro stem-derived biochars: a machine learning-assisted mechanistic elucidation
Source: RSC Adv. 2026 May 6;16(26):23554–84. doi: 10.1039/d6ra01444e (PMC13148361; doi:10.1039/d6ra01444e)
Supplement: RA-016-D6RA01444E-s001 [file RA-016-D6RA01444E-s001.pdf]

Supplementary Information (SI) for RSC Advances.
This journal is © The Royal Society of Chemistry 2026

Supplementary Information (SI)

Title: Comparative immobilization of labile Pb and Zn fractions in contaminated soil using jackfruit seed-, sugarcane bagasse-, and taro stem-derived biochars: A machine learning-assisted mechanistic elucidation.

Table S1. Parameter of ICP-MS Agilent 7900 and recovery values of elements in sediment reference material MESS-4

**Operating conditions of ICP-MS Agilent 7900**			
High-Frequency Power (w)	~1600		
Sampling depth (mm)	~10		
Carrier gas flow rate (L/min)	~0,7		
Auxiliary gas flow rate (L/min)	~0,3		
Peristaltic nebulizer (concentric glass)	MicroMist		
Spray chamber temperature (°C)	2		
Helium gas flow rate (mL/min)	~4,3		
Hydrogen gas flow rate (mL/min)	~4,2		
Peristaltic pump speed	0,1 (0,5 mL/min)		
Internal standard	^{115}In		
Isotopes	^{208}Pb, ^{66}Zn		
LOD and LOQ values of metals using ICP-MS	Element	LOD (ng/L)	LOQ (ng/L)
	Pb	13.7	41.5
	Zn	1.5	4.5
Recovery values of metal elements (%) for MESS-4	Element	Recovery (%)	RSD (%)
	Pb	96.2	9.8
	Zn	91.8	10.4

Table S2. *Two-way ANOVA results for the effects of biochar type and application rate on soil pH, organic carbon (OC), and electrical conductivity (EC) after 1-month incubation.*

**Dependent variable**	**Source of variation**	**df**	***F***	***p***	**Significance**
**pH**	Biochar type	2/24	86.36	$< 1 \times 10^{-12}$	***
	Application rate	3/24	109.16	3.12×10^{-14}	***
	Biochar × Rate	6/24	47.63	$< 1 \times 10^{-15}$	***
**OC (%)**	Biochar type	2/24	86.36	$< 1 \times 10^{-12}$	***
	Application rate	3/24	109.16	3.12×10^{-14}	***
	Biochar × Rate	6/24	28.14	1.84×10^{-11}	***
**EC (µS cm^{-1})**	Biochar type	2/24	77.21	2.06×10^{-12}	***
	Application rate	3/24	75.07	1.12×10^{-12}	***
	Biochar × Rate	6/24	18.57	2.47×10^{-8}	***

Note. *Two-way ANOVA was performed to evaluate the effects of biochar type and application rate on soil chemical properties. *, **, and *** indicate statistical significance at $p < 0.05$, $p < 0.01$, and $p < 0.001$, respectively.*

Table S3. *Distribution of Pb among Tessier sequential extraction fractions (F1-F5) as affected by biochar type and amendment rate (%) (mean ± SD, n = 3).*

Treatment	Rate (%)	%F1	%F2	%F3	%F4	%F5
BS	0	11.9 ± 0.6	46.5 ± 1.2	15.9 ± 0.7	2.9 ± 0.3	22.9 ± 1.0
SB3	3	10.2 ± 0.5	47.5 ± 1.1	16.5 ± 0.6	3.8 ± 0.3	22.1 ± 0.9
SB5	5	8.9 ± 0.4	50.0 ± 1.3	17.3 ± 0.7	4.3 ± 0.4	19.5 ± 0.8
SB10	10	7.9 ± 0.4	53.6 ± 1.5	18.5 ± 0.8	4.9 ± 0.4	15.2 ± 0.7
JB3	3	10.6 ± 0.5	44.0 ± 1.2	16.7 ± 0.7	4.1 ± 0.3	24.7 ± 1.1
JB5	5	9.5 ± 0.5	45.9 ± 1.1	17.5 ± 0.7	4.6 ± 0.4	22.5 ± 0.9
JB10	10	8.4 ± 0.4	49.2 ± 1.3	18.8 ± 0.8	5.5 ± 0.5	18.1 ± 0.8
TB3	3	9.0 ± 0.4	49.4 ± 1.2	16.8 ± 0.7	4.3 ± 0.4	20.6 ± 0.9
TB5	5	7.0 ± 0.3	51.8 ± 1.4	17.6 ± 0.7	5.0 ± 0.4	18.6 ± 0.8
TB10	10	5.1 ± 0.3	55.5 ± 1.6	19.2 ± 0.8	5.9 ± 0.5	14.3 ± 0.7

(*Values are presented as mean ± standard deviation (n = 3). BS represents Pb/Zn contaminated soil without biochar amendment (control soil or blank soil). SB3 - SB10, JB3 - JB10, and TB3 - TB10 indicate contaminated soils amended with 3%, 5%, and 10% (w/w) of the sugarcane bagasse, jackfruit seed, and taro stem biochars, respectively. All samples were incubated for 30 days.*

Table S4. *Results of two-way ANOVA showing the effects of biochar type, application rate, and their interaction on Pb fractions after 30 days of incubation.*

Pb fraction	Source of variation	df	F-value	p-value	Significance
**F1 (exchangeable)**	Biochar type	2/24	48.6	2.98×10^{-9}	***

Pb fraction	Source of variation	df	F-value	p-value	Significance
	Application rate	3/24	312.4	1×10^{-15}	***
	Biochar × Rate	6/24	9.7	5.92×10^{-5}	***
**F2 (carbonate-bound)**	Biochar type	2/24	26.9	2.21×10^{-7}	***
	Application rate	3/24	58.3	4.63×10^{-12}	***
	Biochar × Rate	6/24	4.2	0.00594	**
**F3 (Fe/Mn oxide-bound)**	Biochar type	2/24	18.4	1.15×10^{-5}	***
	Application rate	3/24	44.7	9.64×10^{-11}	***
	Biochar × Rate	6/24	3.9	0.00836	**
**F4 (OM-bound)**	Biochar type	2/24	31.2	6.02×10^{-8}	***
	Application rate	3/24	96.5	1.61×10^{-13}	***
	Biochar × Rate	6/24	5.6	0.00146	**
**F5 (residual)**	Biochar type	2/24	42.1	7.31×10^{-9}	***
	Application rate	3/24	287.9	1×10^{-15}	***
	Biochar × Rate	6/24	11.4	1.74×10^{-5}	***

Notes: *, **, and *** indicate significance at $p < 0.05$, $p < 0.01$, and $p < 0.001$, respectively; ns = not significant.

Table S5. *Distribution of Zn among Tessier sequential extraction fractions (F1-F5) as affected by biochar type and amendment rate (%) (mean ± SD, n = 3).*

Treatment	Rate (%)	%F1	%F2	%F3	%F4	%F5
BS	0	12.9 ± 0.7	23.7 ± 1.0	20.5 ± 0.8	5.2 ± 0.4	37.7 ± 1.3

Treatment	Rate (%)	%F1	%F2	%F3	%F4	%F5
SB3	3	10.7 ± 0.5	24.9 ± 1.1	21.5 ± 0.9	5.1 ± 0.3	37.7 ± 1.2
SB5	5	9.5 ± 0.5	25.7 ± 1.2	22.4 ± 0.9	4.9 ± 0.3	37.5 ± 1.1
SB10	10	8.4 ± 0.4	26.7 ± 1.3	23.5 ± 1.0	4.6 ± 0.3	36.8 ± 1.0
JB3	3	9.5 ± 0.5	25.7 ± 1.1	21.3 ± 0.9	4.5 ± 0.3	39.1 ± 1.4
JB5	5	8.6 ± 0.4	26.3 ± 1.2	22.1 ± 0.9	4.2 ± 0.3	38.7 ± 1.3
JB10	10	7.5 ± 0.4	27.3 ± 1.3	23.3 ± 1.0	3.9 ± 0.2	38.0 ± 1.2
TB3	3	10.1 ± 0.5	25.4 ± 1.1	22.2 ± 0.9	5.6 ± 0.4	36.8 ± 1.1
TB5	5	8.9 ± 0.4	26.1 ± 1.2	23.1 ± 1.0	5.3 ± 0.4	36.6 ± 1.1
TB10	10	7.8 ± 0.4	27.1 ± 1.3	24.4 ± 1.0	5.0 ± 0.3	35.7 ± 1.0

(*Values are presented as mean ± standard deviation (n = 3). BS represents Pb/Zn contaminated soil without biochar amendment (control soil or blank soil). SB3 - SB10, JB3 - JB10, and TB3 - TB10 indicate contaminated soils amended with 3%, 5%, and 10% (w/w) of the sugarcane bagasse, jackfruit seed, and taro stem biochars, respectively. All samples were incubated for 30 days.*

Table S6. *Results of two-way ANOVA showing the effects of biochar type, application rate, and their interaction on Zn fractions after 30 days of incubation.*

Zn fraction	Source of variation	df	F	p-value	Sig
**F1 (exchangeable)**	Biochar type	2/24	36.8	2.21×10^{-8}	***
	Application rate	3/24	214.5	1.01×10^{-15}	***

Zn fraction	Source of variation	df	F	p-value	Sig
	Biochar × Rate	6/24	7.9	1.73×10^{-4}	***
**F2 (carbonate-bound)**	Biochar type	2/24	14.6	6.52×10^{-5}	***
	Application rate	3/24	22.3	1.14×10^{-7}	***
	Biochar × Rate	6/24	3.1	0.0192	*
**F3 (Fe/Mn oxide-bound)**	Biochar type	2/24	19.8	5.03×10^{-6}	***
	Application rate	3/24	41.6	3.27×10^{-10}	***
	Biochar × Rate	6/24	2.8	0.0318	*
**F4 (OM-bound)**	Biochar type	2/24	28.4	1.12×10^{-7}	***
	Application rate	3/24	64.9	2.08×10^{-12}	***
	Biochar × Rate	6/24	4.9	0.0016	**
**F5 (residual)**	Biochar type	2/24	21.7	2.64×10^{-6}	***
	Application rate	3/24	97.5	4.51×10^{-14}	***
	Biochar × Rate	6/24	6.2	4.87×10^{-4}	***

Notes: *, **, and *** indicate significance at $p < 0.05$, $p < 0.01$, and $p < 0.001$, respectively; ns = not significant.

Table S7. *Machine learning input dataset for predicting the distribution of Pb and Zn among geochemical fractions (F1–F5) in biochar-amended soils. The dataset includes soil physicochemical properties (pH, organic carbon, and electrical conductivity), biochar characteristics (type, application rate, and pH), and metal type as predictor variables.*

Nr	Sample	Biochar	Rate (%)	Metal	pH_soil	OC_soil (%)	EC_soil (μS/cm)	F1 (%)	F2 (%)	F3 (%)	F4 (%)	F5 (%)
1	BS	None	0	Pb	6.84	2.36	52	11.9	46.5	15.9	2.9	22.9
2	BS	None	0	Zn	6.84	2.36	52	12.9	23.7	20.5	5.2	37.7
3	SB3	SB	3	Pb	7.02	2.92	76	10.2	47.5	16.5	3.8	22.1
4	SB3	SB	3	Zn	7.02	2.92	76	10.7	24.9	21.5	5.1	37.7
5	SB5	SB	5	Pb	7.16	3.28	98	8.9	50.0	17.3	4.3	19.5
6	SB5	SB	5	Zn	7.16	3.28	98	9.5	25.7	22.4	4.9	37.5
7	SB10	SB	10	Pb	7.34	3.82	138	7.9	53.6	18.5	4.9	15.2
8	SB10	SB	10	Zn	7.34	3.82	138	8.4	26.7	23.5	4.6	36.8
9	JB3	JB	3	Pb	7.08	2.78	74	10.6	44.0	16.7	4.1	24.7
10	JB3	JB	3	Zn	7.08	2.78	74	9.5	25.7	21.3	4.5	39.1
11	JB5	JB	5	Pb	7.20	3.02	96	9.5	45.9	17.5	4.6	22.5
12	JB5	JB	5	Zn	7.20	3.02	96	8.6	26.3	22.1	4.2	38.7
13	JB10	JB	10	Pb	7.36	3.46	132	8.4	49.2	18.8	5.5	18.1
14	JB10	JB	10	Zn	7.36	3.46	132	7.5	27.3	23.3	3.9	38.0
15	TB3	TB	3	Pb	7.15	2.60	118	9.0	49.4	16.8	4.3	20.6
16	TB3	TB	3	Zn	7.15	2.60	118	10.1	25.4	22.2	5.6	36.8
17	TB5	TB	5	Pb	7.15	2.60	165	7.0	51.8	17.6	5.0	18.6
18	TB5	TB	5	Zn	7.15	2.60	165	8.9	26.1	23.1	5.3	36.6
19	TB10	TB	10	Pb	7.55	3.05	240	5.1	55.5	19.2	5.9	14.3
20	TB10	TB	10	Zn	7.55	3.05	240	7.8	27.1	24.4	5.0	35.7

Table S8. *Input dataset for machine learning models predicting Pb concentrations (mg kg⁻¹) in geochemical fractions (F1–F5) based on soil properties and biochar application parameters.*

Sample	Biochar	Rate (%)	pH_soil	OC_soil (%)	EC_soil (μS/cm)	F1 (mg/kg)	F2 (mg/kg)	F3 (mg/kg)	F4 (mg/kg)	F5 (mg/kg)
BS	None	0	6.84	2.36	52	464.6	1822.9	622.6	113.4	896.5
SB3	SB	3	7.02	2.92	76	387.5	1810.4	630.1	144.3	842.7
SB5	SB	5	7.16	3.28	98	331.8	1862.9	644.5	159.6	727.2
SB10	SB	10	7.34	3.82	138	280.6	1909.8	659.7	174.6	541.4
JB3	JB	3	7.08	2.78	74	402.1	1676.3	634.9	155.8	939.1
JB5	JB	5	7.20	3.02	96	354.6	1712.8	651.6	171.9	840.6
JB10	JB	10	7.36	3.46	132	299.4	1756.2	670.1	194.7	645.5
TB3	TB	3	7.15	2.60	118	343.8	1882.7	639.4	162.6	783.5
TB5	TB	5	7.15	2.60	165	260.5	1932.4	657.8	187.9	692.4
TB10	TB	10	7.55	3.05	240	182.6	1979.8	684.9	211.6	509.2

Table S9. *Input dataset for machine learning models predicting Zn concentrations (mg kg⁻¹) in geochemical fractions (F1–F5) based on soil properties and biochar application parameters.*

Sample	Biochar	Rate (%)	pH_soil	OC_soil (%)	EC_soil (μS/cm)	F1 (mg/kg)	F2 (mg/kg)	F3 (mg/kg)	F4 (mg/kg)	F5 (mg/kg)
BS	None	0	6.84	2.36	52	230.5	424.6	366.7	93.1	675.1

Sample	Biochar	Rate (%)	pH_soil	OC_soil (%)	EC_soil (µS/cm)	F1 (mg/kg)	F2 (mg/kg)	F3 (mg/kg)	F4 (mg/kg)	F5 (mg/kg)
SB3	SB	3	7.02	2.92	76	185.6	432.7	374.5	89.3	655.8
SB5	SB	5	7.16	3.28	98	162.3	438.1	382.9	83.4	641.2
SB10	SB	10	7.34	3.82	138	139.8	444.5	391.6	76.9	612.4
JB3	JB	3	7.08	2.78	74	158.4	429.6	356.1	74.8	653.2
JB5	JB	5	7.20	3.02	96	142.6	435.2	364.7	69.5	640.1
JB10	JB	10	7.36	3.46	132	121.3	441.8	375.9	62.4	614.6
TB3	TB	3	7.15	2.60	118	176.9	446.3	391.4	98.5	646.8
TB5	TB	5	7.15	2.60	165	154.2	451.7	399.6	91.2	633.1
TB10	TB	10	7.55	3.05	240	131.6	458.4	412.8	83.7	602.9

Table S10. Performance of machine learning models for predicting Pb and Zn concentrations in different Tessier fractions.

Metal	Fraction	Model	RMSE	MAE	R^2
Pb	F1	RF	36.88	30.48	0.776
	F1	XGB	**34.60**	**28.68**	**0.800**
	F2	RF	118.85	96.72	0.045
	F2	XGB	139.13	116.91	0.039
	F3	RF	**28.78**	**22.24**	**0.240**
	F3	XGB	32.57	25.89	0.185
	F4	RF	18.64	14.80	0.621
	F4	XGB	**18.32**	**14.09**	**0.658**
	F5	RF	52.51	47.68	0.843
	F5	XGB	**50.69**	**39.34**	**0.849**
Zn	F1	RF	**23.62**	**18.59**	**0.556**
	F1	XGB	26.75	22.23	0.474
	F2	RF	26.68	21.41	0.074
	F2	XGB	**23.94**	**18.81**	**0.255**
	F3	RF	27.25	**20.92**	0.134
	F3	XGB	27.26	21.93	**0.203**
	F4	RF	8.77	7.72	0.429
	F4	XGB	**8.43**	**7.03**	**0.474**
	F5	RF	**17.96**	**14.54**	**0.409**
	F5	XGB	20.60	16.98	0.292

Note: *RF denotes Random Forest, and XGB denotes Extreme Gradient Boosting. RMSE (root mean square error) and MAE (mean absolute error) represent model prediction errors, with lower values indicating higher predictive accuracy. The coefficient of determination (R^2) reflects the proportion of variance in the observed data explained by the model, where values closer to 1 indicate superior model performance. F1-F5 correspond to the chemical fractions obtained using the Tessier sequential extraction procedure: F1, exchangeable fraction; F2, carbonate-bound fraction; F3, Fe-Mn oxide-bound fraction; F4, organic matter/sulfide-bound fraction; and F5, residual fraction. Values shown in* ***bold*** *indicate the best-performing model for each metal fraction, based on a combined evaluation of RMSE, MAE, and R^2.* dataset: 30 observations**.**

Table 11. Feature importance of environmental variables controlling Pb distribution among Tessier fractions (F1–F5) predicted by RF and XGB models

(a) Random Forest (RF)

Fraction	Rank	Feature	Importance score
**F1**	1	EC	52 734.35
	2	pH	46 459.18
	3	Rate	31 747.65
	4	OC	26 656.74
**F2**	1	EC	103 461.67
	2	pH	90 992.84
	3	OC	77 415.18
	4	Rate	34 739.26
**F3**	1	pH	6 986.82
	2	EC	6 918.78
	3	OC	6 541.76
	4	Rate	4 923.19
**F4**	1	pH	6 538.26
	2	EC	6 389.89
	3	OC	6 073.56
	4	Rate	5 003.90
**F5**	1	EC	134 917.88
	2	pH	121 481.27
	3	Rate	116 383.43
	4	OC	88 274.38

Feature importance scores were obtained from Random Forest (RF) (mean decrease in impurity) and XGBoost (XGB) (gain). Larger values indicate stronger contribution to Pb fraction prediction.

(b) XGBoost (XGB)

Fraction	Rank	Feature	Gain
**F1**	1	EC	0.677
	2	pH	0.242
	3	OC	0.072
	4	Rate	0.009
**F2**	1	EC	0.387
	2	pH	0.344
	3	OC	0.245
	4	Rate	0.024
**F3**	1	pH	0.445
	2	OC	0.249

Fraction	Rank	Feature	Gain
	3	EC	0.237
	4	Rate	0.069
**F4**	1	pH	0.420
	2	Rate	0.237
	3	EC	0.175
	4	OC	0.168
**F5**	1	EC	0.489
	2	pH	0.394
	3	OC	0.091
	4	Rate	0.026

Table 12. Feature importance of environmental variables controlling Zn distribution among Tessier fractions (F1–F5) predicted by RF and XGB models

Feature importance scores were derived from Random Forest (RF) (mean decrease in impurity) and XGBoost (XGB) (gain). Higher values indicate stronger influence on Zn fraction prediction.

(a) Random Forest (RF)

Fraction	Rank	Feature	Importance score
**F1**	1	pH	8866.85
	2	OC	8616.17
	3	EC	8094.50
	4	Rate	7130.26
**F2**	1	EC	6647.00
	2	OC	4744.27
	3	pH	3916.44
	4	Rate	1631.55
**F3**	1	EC	7237.76
	2	pH	5383.37
	3	OC	4761.08
	4	Rate	2456.18
**F4**	1	OC	1108.34
	2	pH	853.29
	3	EC	717.26
	4	Rate	598.46
**F5**	1	pH	3897.79
	2	OC	3481.12
	3	EC	3124.13
	4	Rate	2992.66

(b) XGBoost (XGB)

Fraction	Rank	Feature	Gain
**F1**	1	pH	0.421
	2	Rate	0.273
	3	OC	0.253
	4	EC	0.053
**F2**	1	EC	0.494
	2	pH	0.250
	3	OC	0.229
	4	Rate	0.027
**F3**	1	EC	0.457
	2	pH	0.282
	3	OC	0.249
	4	Rate	0.011
**F4**	1	OC	0.606
	2	pH	0.212
	3	EC	0.128
	4	Rate	0.053

Fraction	Rank	Feature	Gain
**F5**	1	OC	0.330
	2	pH	0.324
	3	EC	0.219
	4	Rate	0.128

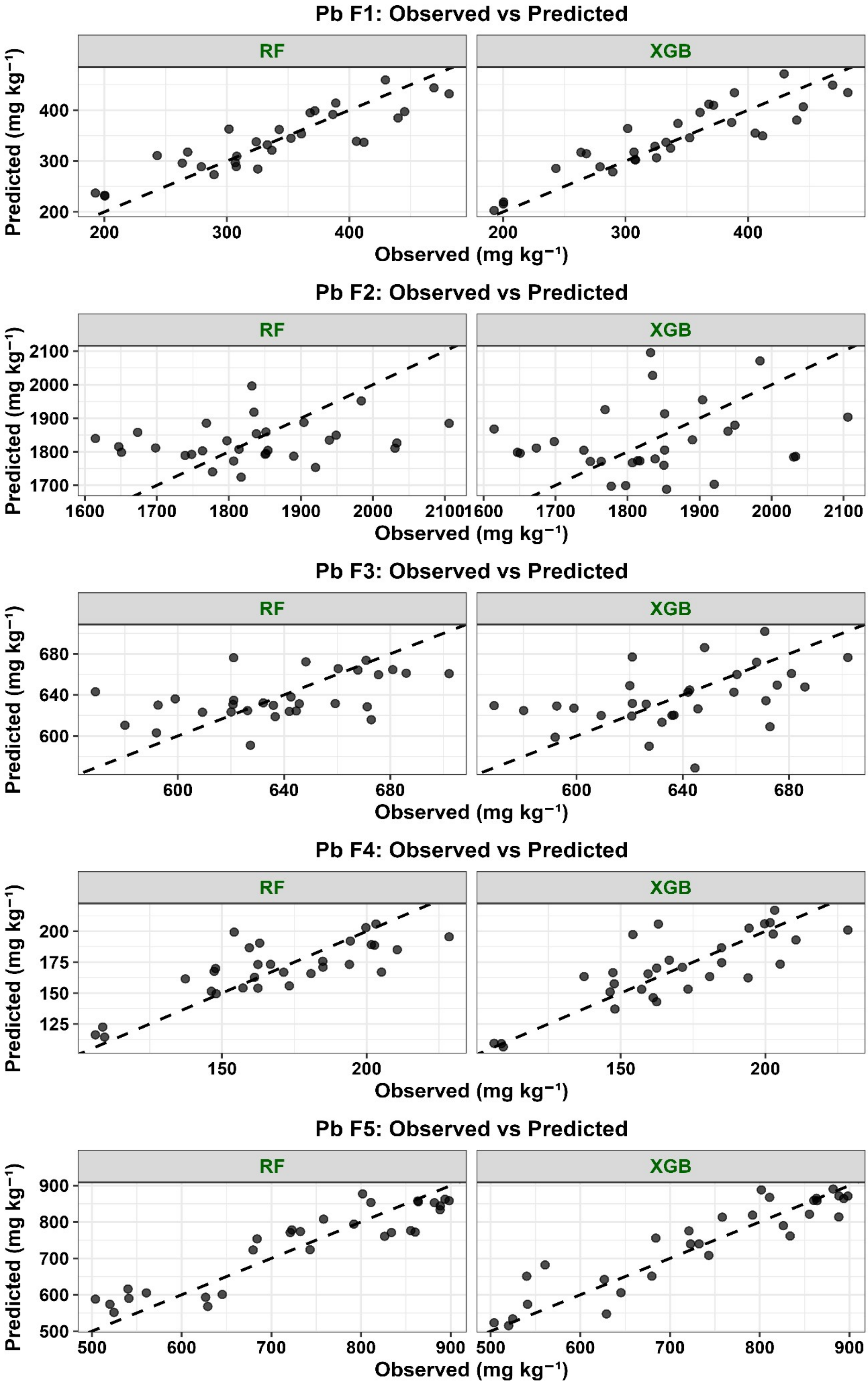

Figure S1. Observed and predicted of Pb fractions (F1-F5)

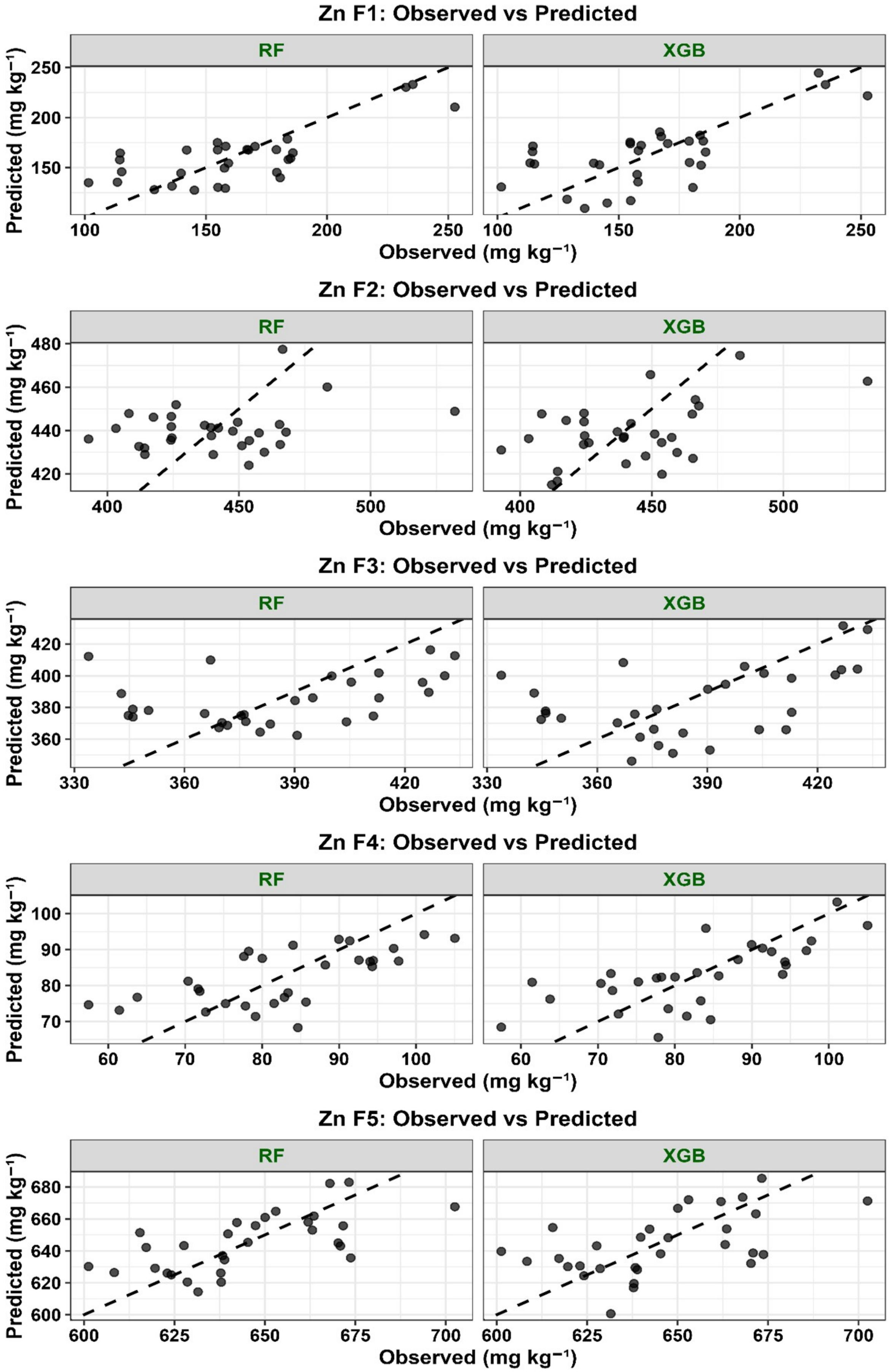

Figure S2. Observed and predicted of Zn fractions (F1-F5)

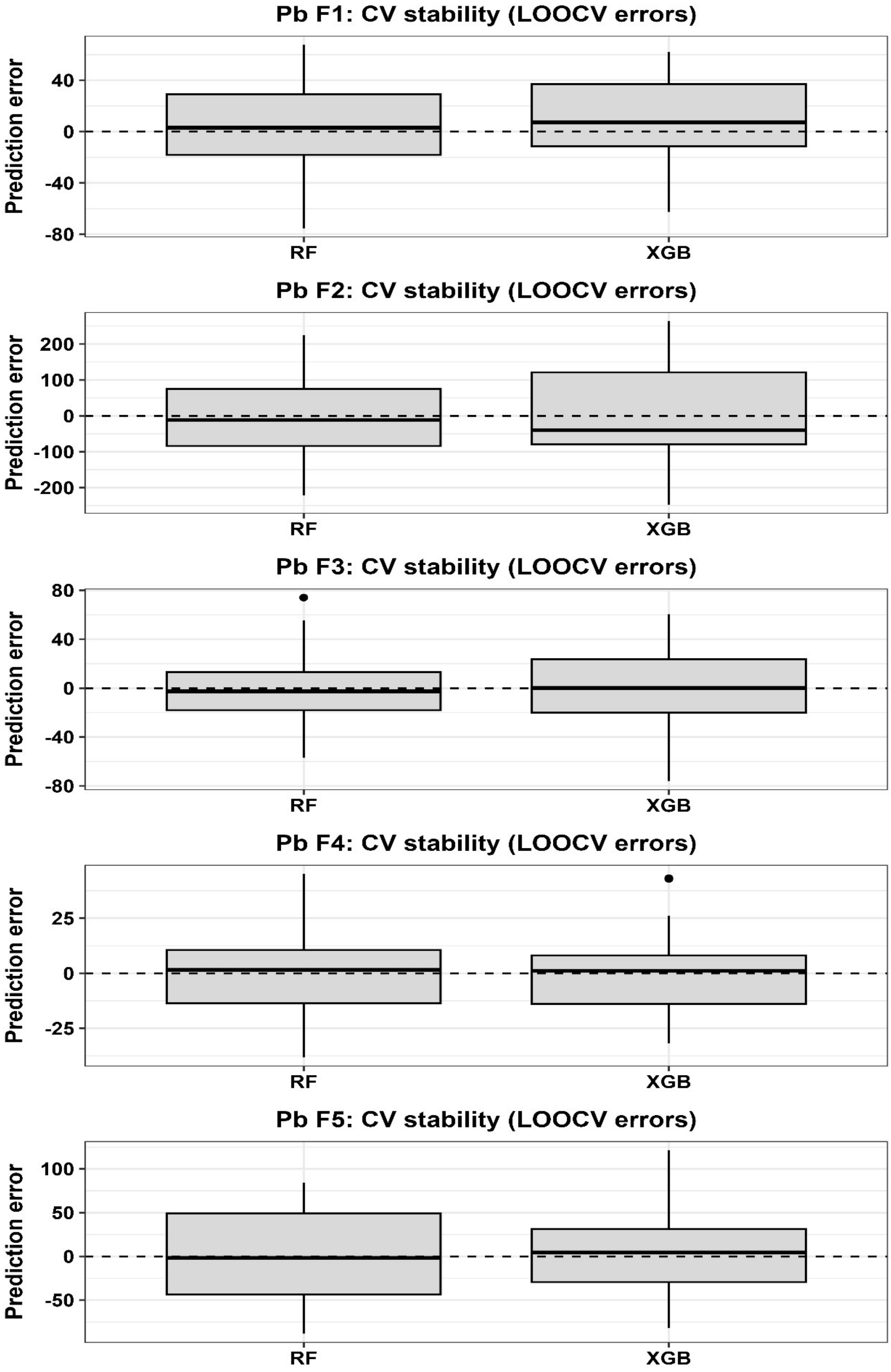

Figure S3. CV stability of Pb fractions (F1-F5)

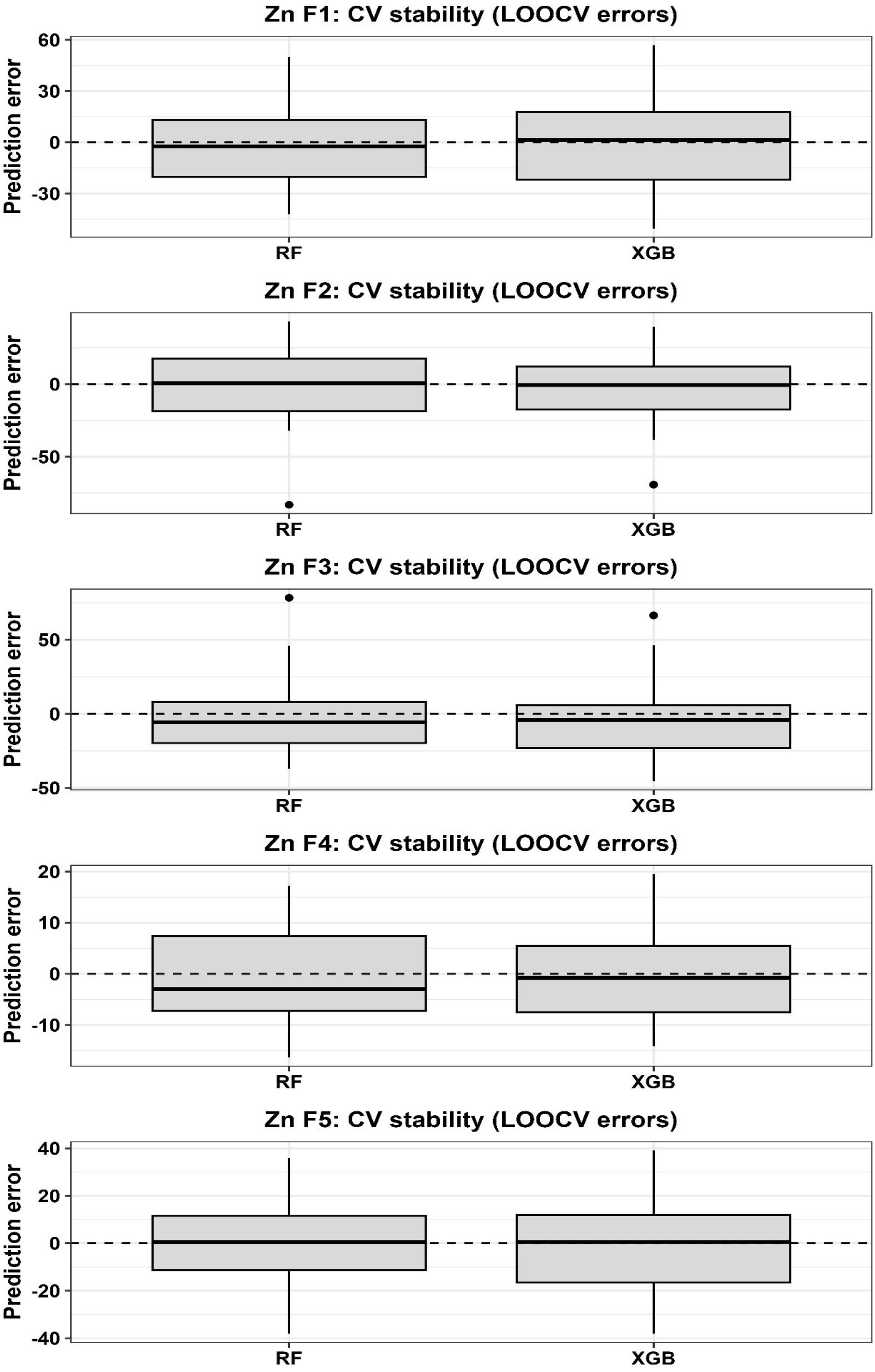

Figure S4. CV stability of Zn fractions (F1-F5)

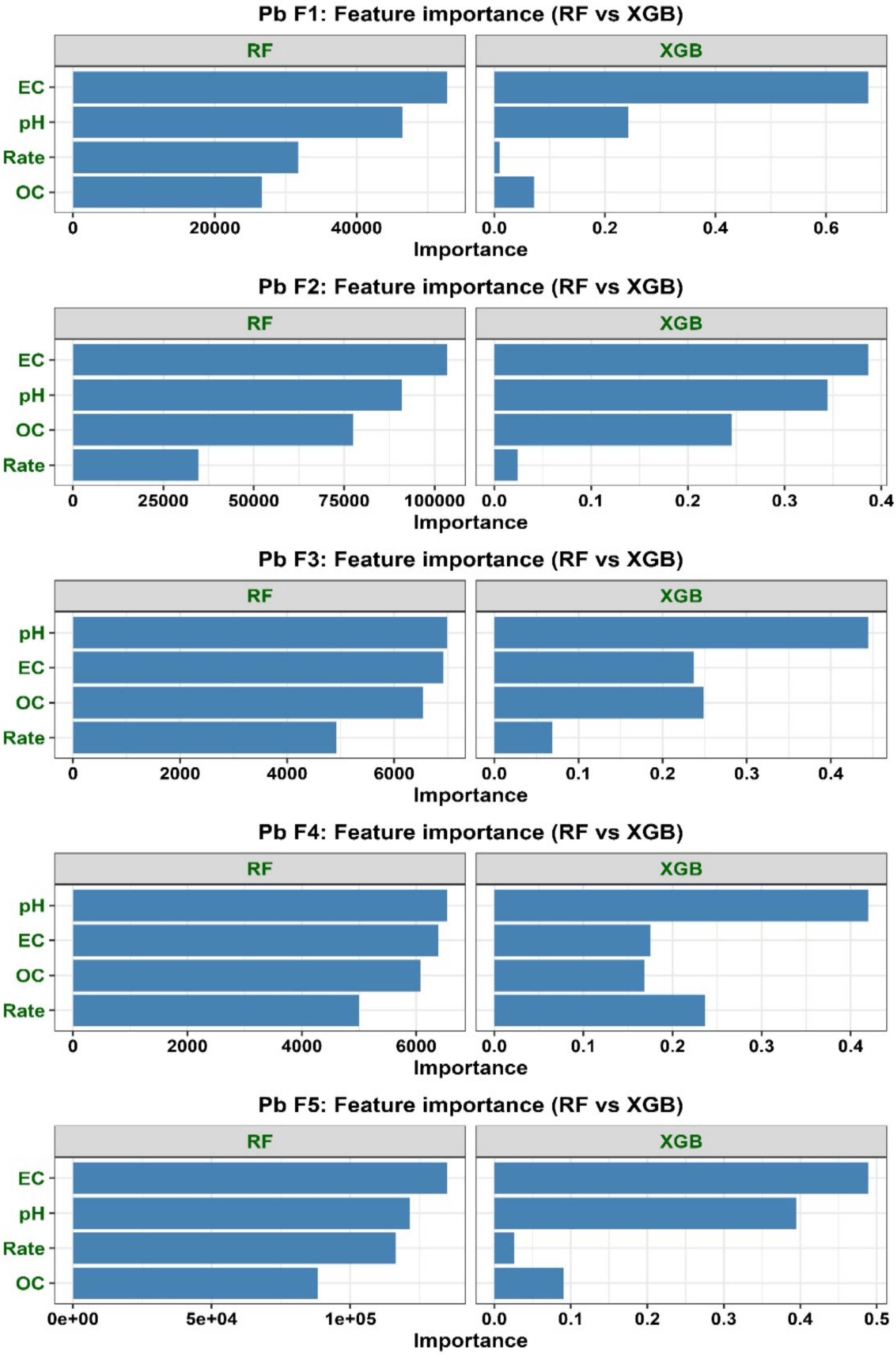

Figure S5. Importance of Pb fractions (F1-F5)

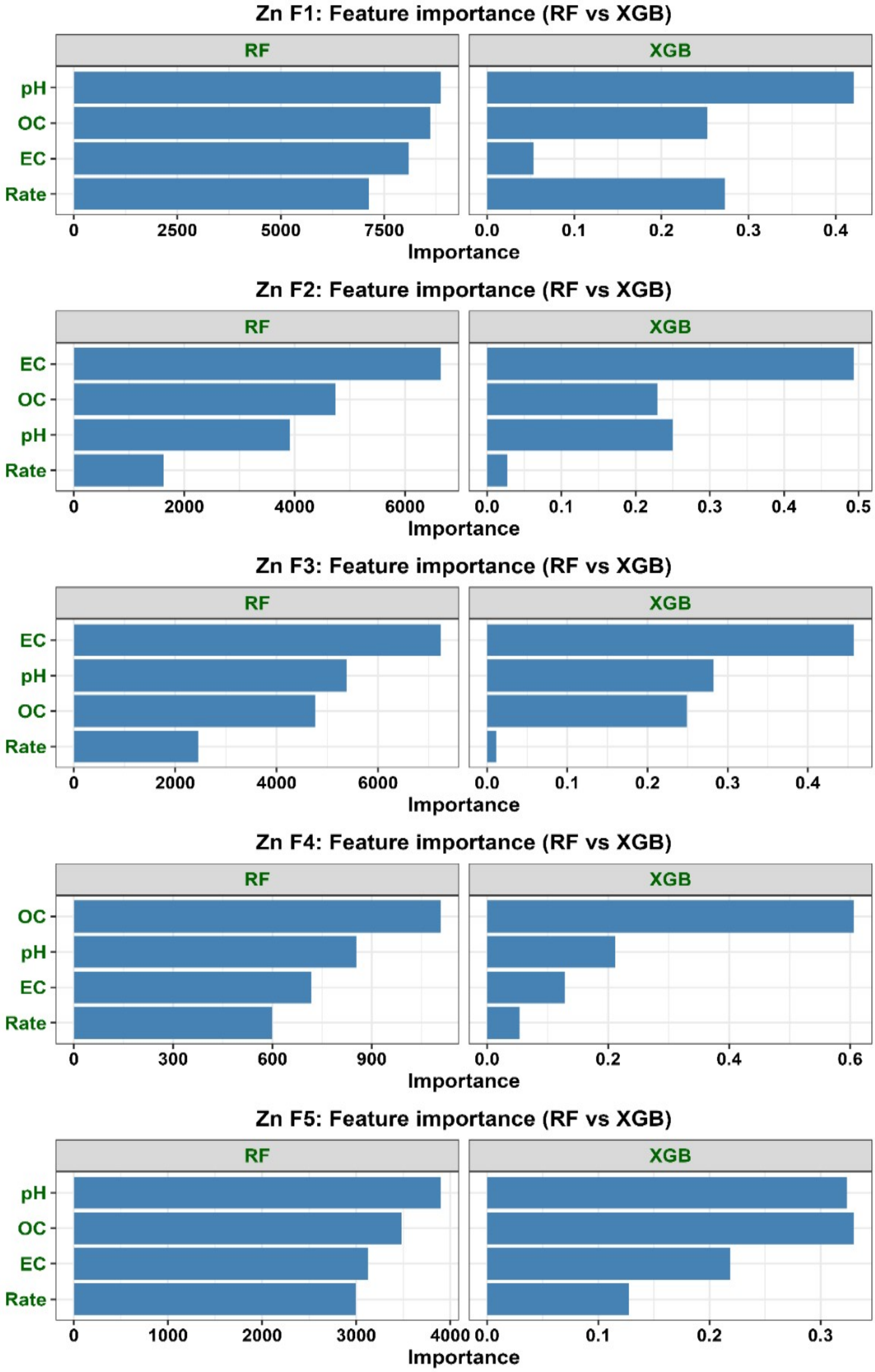

Figure S6. Importance of Zn fractions (F1-F5)
